# IFNγ-inducible Gbp4 and Irgb6 contribute to experimental cerebral malaria pathology in the olfactory bulb

**DOI:** 10.1128/mbio.01249-25

**Published:** 2025-07-03

**Authors:** Julia Matsuo-Dapaah, Jalal Alshaweesh, Michelle Sue Jann Lee, Tomoya Hayashi, Rashmi Dash, Masafumi Kuroda, Kazuki Tainaka, Manabu Ozawa, Ayumi Kuratani, Masahiro Yamamoto, Kaiwen Liu, Ryutaro Fukui, Kensuke Miyake, Kouji Kobiyama, Laurent Rénia, Ken J. Ishii, Cevayir Coban

**Affiliations:** 1 Division of Malaria Immunology, Department of Microbiology and Immunology, The Institute of Medical Science (IMSUT), The University of Tokyo 592629 , Tokyo, Japan; 2 Graduate School of Medicine, The University of Tokyo 13143, Tokyo, Japan; 3 Division of Vaccine Science, Department of Microbiology and Immunology, The Institute of Medical Science (IMSUT), The University of Tokyo 592714 , Tokyo, Japan; 4 International Vaccine Design Center (VDesC), The Institute of Medical Science (IMSUT), The University of Tokyo 26430 , Tokyo, Japan; 5 Graduate School of Frontier Sciences, The University of Tokyo 105219 , Tokyo, Japan; 6 WPI International Research Center for Neurointelligence (IRCN), University of Tokyo 578447 , Tokyo, Japan; 7 Department of Cell Biology, Graduate School of Medicine, The University of Tokyo 13143, Tokyo, Japan; 8 Department of System Pathology for Neurological Disorders, Center for Bioresources, Brain Research Institute, Niigata University 220888, Niigata, Japan; 9 Laboratory for Synthetic Biology, RIKEN Center for Biosystems Dynamics Research 208578, Osaka, Japan; 10 Laboratory of Reproductive Systems Biology, Institute of Medical Science (IMSUT), The University of Tokyo 596892 , Tokyo, Japan; 11 Core Laboratory for Developing Advanced Animal Models, Center for Experimental Medicine and Systems Biology, University of Tokyo 13143, Tokyo, Japan; 12 Department of Immunoparasitology, WPI Immunology Frontier Research Center (IFReC), Osaka University 199485 , Osaka, Japan; 13 Division of Infectious Genetics, Department of Microbiology and Immunology, The Institute of Medical Science (IMSUT), The University of Tokyo 592632 , Tokyo, Japan; 14 Lee Kong Chian School of Medicine, Nanyang Technological University 54761, Singapore, Singapore; 15 School of Biological Sciences, Nanyang Technological University 219572, Singapore, Singapore; 16 A*STAR Infectious Diseases Labs, Agency for Science, Technology, and Research (A*STAR) 54759, Singapore, Singapore; 17 The University of Tokyo Pandemic Preparedness, Infection and Advanced Research Center (UTOPIA), The University of Tokyo 13143, Tokyo, Japan; The George Washington University Milken Institute of Public Health, Washington, DC, USA

**Keywords:** cerebral malaria, GTPases, olfactory bulb, Gbp4, Irgb6

## Abstract

Cerebral malaria (CM) is a severe and often fatal complication of *Plasmodium falciparum* infection. Although much progress has been made in understanding CM, the precise pathogenesis remains elusive. The olfactory bulb (OB) has emerged as a critical site of immunopathology in experimental cerebral malaria (ECM) models, but its contribution to disease progression is not fully understood. To investigate the molecular mechanisms driving early ECM pathogenesis, we conducted transcriptomic profiling of the OB to identify key genes associated with disease onset. Our analysis revealed significant early upregulation of interferon (IFN)-inducible GTPases, particularly Irgb6 and Gbp4, effectors downstream of IFN-γ but not IFN-α/β signaling, suggesting their involvement in ECM pathology. Using *Gbp4^−/−^*, *Irgb6*^*−/−*^, and double knockout (*Irgb6^−/−^ Gbp4*^*−*/*−*^) mice, we identified a pathological role for these GTPases. Mechanistically, we found that double-knockout mice exhibited increased infiltration of CD4^+^ and CD8^+^ T cells into the brain but with reduced T cell functionality and impaired antigen presentation by endothelial cells, leading to enhanced parasite accumulation in the OB. This disruption in immune regulation ultimately conferred improved survival in the *Irgb6^−/−^ Gbp4*^*−*/*−*^ mice and indicated the pathological impact of Gbp4 and Irgb6 in ECM. These findings reveal that Gbp4 and Irgb6 play important roles in the early immunopathogenesis of ECM by modulating antigen processing and presentation in the OB, thereby shaping immune cell dynamics. Our work shows the dual role of Irgb6 and Gbp4 GTPases in host defence and immunopathology and offers new insights into ECM mechanisms and antigen presentation.

**IMPORTANCE:**

Cerebral malaria (CM) arises from an excessive inflammatory response and blood-brain-barrier (BBB) dysfunction in *Plasmodium*-infected hosts, but the precise mechanisms driving early-stage pathogenesis remain unclear. Through RNA sequencing of the olfactory bulb (OB) in a murine experimental cerebral malaria (ECM) model, we identified the early upregulation of interferon (IFN)-inducible GTPases, Irgb6 and Gbp4, key effectors downstream of IFN-γ signaling. Our results demonstrate that Gbp4 and Irgb6 synergistically contribute to ECM pathology by regulating antigen cross-presentation in endothelial cells. This dysregulation leads to abnormal parasite burden and alters the accumulation of CD4+ and CD8+ T cells in the brain via the OB, further perturbing inflammation. Our findings suggest a novel mechanism in CM and emphasize the pivotal roles of Gbp4 and Irgb6 in promoting cell-autonomous immune responses that, in turn, escalate pathological inflammation. Our study offers insights into how dysregulated immune responses drive CM progression and suggests potential therapeutic targets to mitigate fatal outcomes.

## INTRODUCTION

Severe malaria is a critical, life-threatening condition that requires immediate attention. In 2023, malaria caused an estimated 597,000 deaths, with a substantial proportion of these fatalities attributed to cerebral malaria (CM), a severe form of *Plasmodium falciparum* infection that primarily affects children under the age of five (1). CM presents as an acute cerebrovascular encephalopathy with unarousable coma, and it is associated with the sequestration of the infected red blood cells (iRBCs) to the brain microvasculature via cytoadherence and the resultant mechanical obstruction of microvessels and/or activation of the brain endothelial cells, inflammation, and blood-brain barrier (BBB) dysregulation (2–4). Dysfunction of the BBB leads to vasogenic cerebral oedema with increased intracranial pressure, herniation, and ischemia, which ultimately leads to mortality in 15%–25% of the cases (5–7). The precise mechanisms involved in the pathogenesis of CM are not fully understood.

The *Plasmodium berghei* ANKA (*Pb*A) infection in C57BL/6 mice, which recapitulates most of the features of human CM, has been used as an experimental model of cerebral malaria (experimental cerebral malaria, ECM) (8). ECM mice show BBB disruption and neurological symptoms, as in the human CM. Infiltration of immune cells, especially T cells, has been observed in the brains of *Pb*A-infected mice, and CD8^+^ T cells, as well as interferon-γ (IFN-γ), has been shown to be essential for the development of ECM (9–11). Cross-presentation of parasite antigens by the brain endothelial cells to cytotoxic CD8^+^ T cells and the secretion of perforins and granzymes by the CD8^+^ T cells induce BBB disruption and subsequent death of mice during ECM (12). A recent study additionally reported the presence of CD3^+^ CD8^+^ T cells in the brain vasculature of children who died from CM (7), suggesting the potential involvement of these cells in human CM as well.

We previously demonstrated that *Pb*A parasites preferentially accumulate in the olfactory bulb (OB) of infected mice, causing both physical and functional damage to this region (13, 14). Additionally, we and others have shown that BBB disruption initiates in the OB (13, 15), making it one of the locations most affected in ECM (16–18). Given that ECM pathology begins in the OB before affecting other areas of the brain, we aimed to investigate the early events originating in the OB that initiate and contribute to ECM pathology.

Here, to explore the early markers of ECM in the OB, we performed transcriptome analysis at early and late stages of ECM and identified *Gbp4* and *Irgb6* as early genes induced as early as day 3 after infection. Gbp4 and Irgb6 are interferon-inducible GTPases, belonging to the p47 immunity-related GTPase (IRG) and p65 guanylate-binding protein (GBP) families, respectively (19–21). IFN-γ can activate anti-microbial cell-autonomous immunity programs involving these GTPases, which have been shown to protect against viral, protozoan, and bacterial infections (21, 22). *Gbp4* expression has been previously observed in macrophages and dendritic cells following infection with Sendai virus or upon IFN-γ stimulation (23, 24). *Irgb6* is more extensively characterized, with its expression detected in macrophages during *Toxoplasma gondii* infection, as well as in T cells and astrocytes, particularly upon IFN-γ stimulation (25–27). Irgb6 has been shown to accumulate on the *T. gondii* parasitophorous vacuole membrane (PVM), along with other IRGs such as Irga6 and Irgd, as well as with GBPs, leading to PVM disruption and parasite death (28–30). Here, we examined the contribution of *Gbp4* and *Irgb6* on the pathogenesis of ECM, by using *Gbp4* deficient (*Gbp4^−/−^*), *Irgb6* deficient (*Irgb6*^−/−^), as well as *Irgb6 Gbp4* double-deficient (*Irgb6^−/−^ Gbp4*^−/−^) mice and found that *Irgb6* and *Gbp4* in response to *Pb*A-mediated early IFN-γ signals contribute to ECM pathology.

## RESULTS

### Transcriptomic profiling of the OB reveals early upregulation of interferon-inducible GTPases *Gbp4* and *Irgb6* during ECM

**Fig 1:**
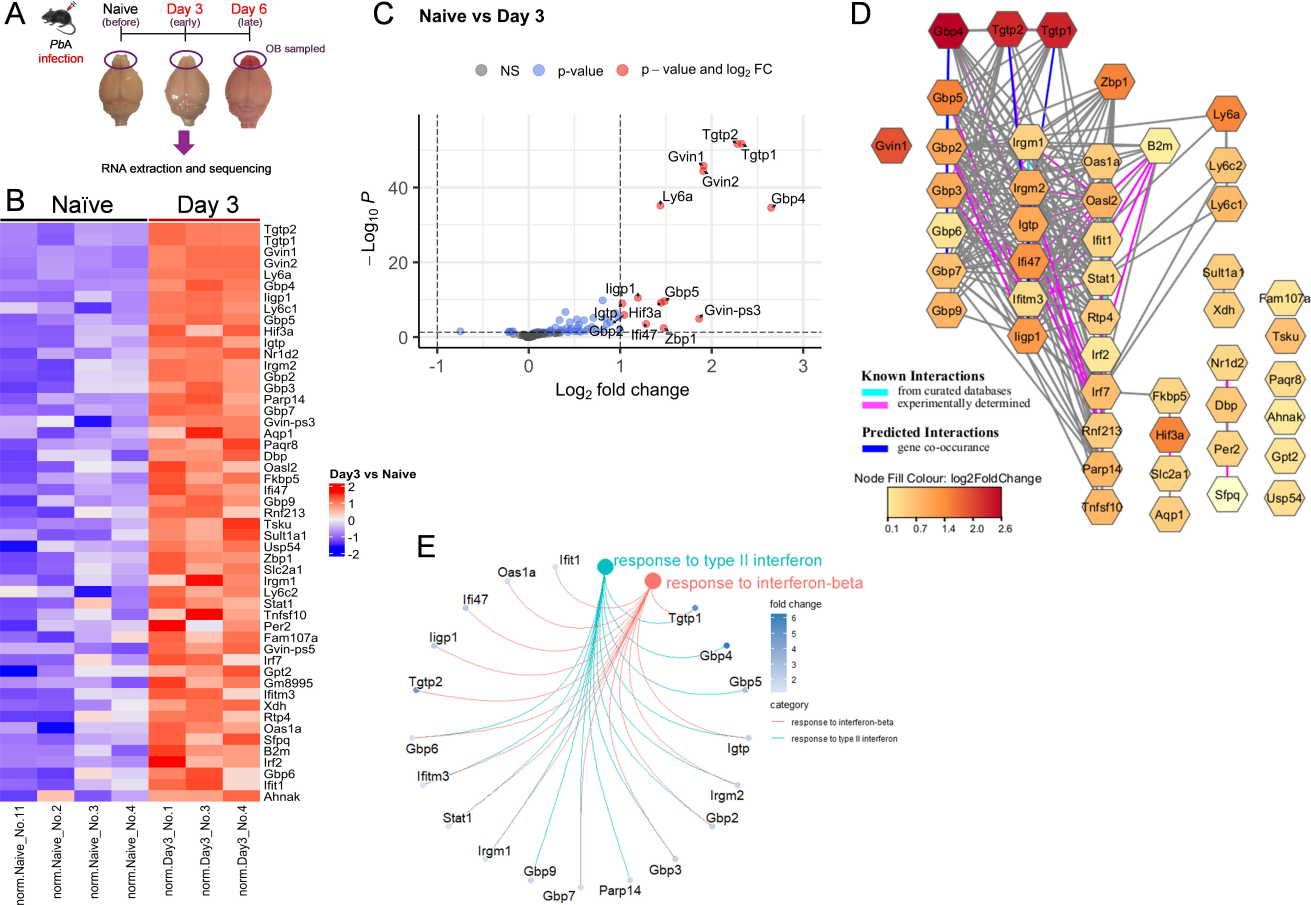
Transcriptomic analysis of the olfactory bulb identifies *Gbp4* and *Irgb6* as interferon-inducible GTPases upregulated early during ECM. (**A**) Schematic outline of the experimental design. Olfactory bulbs were collected at different time points after *Pb*A infection for RNA extraction, sequencing, and transcriptomic analysis (*n* = 3–4 mice/group on days 0, 3, and 6 post-infection). (**B**) Heatmap of differentially expressed genes in the OBs of uninfected versus *Pb*A-infected mice on day 3 post-infection. Fifty-one genes were significantly upregulated, and five genes were downregulated on day 3 compared to uninfected samples (*P*_adj_ < 0.05). (**C**) Volcano plot of differentially expressed genes in the OBs of uninfected versus *Pb*A-infected mice on day 3 post-infection. Significantly upregulated genes (fold-change ≥ 1.0 and adjusted *P*-value < 0.05) are indicated on the upper right (red dots). Horizontal dotted line indicates the cut-off of adjusted *P*-values. Vertical dotted line indicates the cut-off of log_2_ fold-change (−1.0 to 1.0). (**D**) STRING association network of differentially expressed genes upregulated on day 3 post*-Pb*A infection compared to naïve. Node fill color indicates log_2_ fold-change. Edges depict interactions, with aqua for known interactions from curated databases, lavender for experimentally determined known interactions, and blue for predicted gene co-occurrence. (**E**) Gene concept network of two gene ontology biological processes (GO:BP) terms “response to type II interferon” and “response to interferon-beta” enriched in genes differentially upregulated on day 3 post*-Pb*A infection compared to naïve.

To identify early molecular signatures that contribute to the pathology of ECM, we performed sequencing of RNA isolated from OB samples of *Pb*A-infected mice at three time points: day 0 (naïve), day 3 (early infection), and day 6 (late infection) (Fig. 1A). At the late stage of ECM (day 6), 9,136 genes were differentially expressed, with 4,717 genes upregulated and 4,419 downregulated. Table S1 shows genes with |log_2_ fold-change| ≥ 1.0 and *P*_adj_ ≤ 0.05 after the Benjamini-Hochberg procedure was applied to control the false discovery rate (FDR) at 0.05 (31, 32). The majority of upregulated genes on day 6 were linked to the inflammatory and immune response functions, while downregulated genes were predominantly associated with nervous system and neural function (Fig. S1A through C). These findings support and reflect the previous reports of inflammation in the brain at the late stage of ECM, with increased infiltration of immune cells as well as production of chemokines and cytokines and neuronal death (33, 34). We also found that genes related to olfactory G protein-coupled receptor (GPCR) signaling and development were significantly downregulated on day 6 (Fig. S3A through C). In contrast, only 56 genes were differentially expressed on day 3 compared to naïve controls (*P*_adj_ ≤ 0.05), with 14 of these showing significant upregulation (log_2_ fold-change > 1.0) (Fig. 1B and C; Table S2). Notably, *Gbp4, Gvin1, Gvin2, Ly6a, Tgtp1*, and *Tgtp2* were among the most significantly upregulated genes, clustered on the top right of the volcano plot, with both high log_2_ fold-change and low adjusted *P*-values (Fig. 1C). Other significantly upregulated genes included *Gbp2, Gbp5, Gvin-ps3, Hif3a, Ifi47, Igtp, Iigp1*, and *Zbp1*, though these had lower log_2_ fold-changes and higher adjusted *P*-values. Functional annotation with gene ontology (GO) biological process analysis revealed that most of the significantly upregulated genes on day 3 were involved in the innate immune response (*Tgtp1, Tgtp2, Zbp1, Gbp2, Gbp4, Gbp5, Igtp, Ifi47, Iigp1*). Additionally, a high proportion of the upregulated genes belonged to the interferon-inducible GTP-binding protein family, including *Gvin1, Gvin2, Tgtp1, Tgtp2, Gbp2, Gbp4, Gbp5, Igtp, Ifi47,* and *Iigp1*. These genes were further subclassified into IRGs (*Tgtp1* [also known as *Irgb6*], *Tgtp2* [also known as *Irgb6*], *Igtp* [also known as *Irgm3*], *Ifi47* [also known as *Irgd*], and *Iigp1* [also known as *Irga6*]), GVINs (*Gvin1* and *Gvin2*), and GBPs (*Gbp2*, *Gbp4*, and *Gbp5*). Notably, *Tgtp1* and *Tgtp2* both encode for the same protein, Irgb6, and will be referred to as *Irgb6* henceforth. Many of these genes were also upregulated at day 6 post-infection (Fig. S1A) and included known interferon-stimulated genes (ISGs) such as *Oas1a*, *Oasl2*, *Ifit1*, *Rtp4*, *Ifitm3*, and *Irf7* as well as proteasome components, especially immunoproteasome genes such as *Psmb8 (Lmp7), Psmb9 (Lmp2),* and *Psmb10 (Mecl1*) and olfactory receptor signaling pathways (Fig. S2A through C, S3B). The STRING (Search Tool for the Retrieval of Interacting Genes/Proteins) interaction network was utilized to predict protein-protein interactions associated with the day 3 upregulated gene signature (Fig. 1D). *Gbp4* and *Tgtp1/Tgtp2* (*Irgb6*) were central nodes with extensive connections to other proteins in the network, showing the largest log_2_ fold-changes. Other GBPs (*Gbp2, Gbp3, Gbp5, Gbp6, Gbp7, Gbp9*) and IRGs (*Irgm1, Irgm2, Igtp, Ifi47, Ifitm3, Iigp1*) were also identified, with experimentally determined and predicted connections linking them to ISGs (e.g., *Zbp1, Oas1a, Oasl2, Ifit1, Stat1, Rtp4, Irf3, Irf7, Rnf21, Parp14, Tnfsf10*) and MHC-I activation (*B2m*). Additionally, a network of genes related to biological rhythms (*Dbp, Nr1d2, Per2, Sfpq*) was observed among the day 3 upregulated genes.

Furthermore, gene set enrichment analysis of the upregulated genes on day 3 revealed a significant enrichment in pathways associated with the “response to type II interferon” and “response to interferon-beta.” Several genes, including *Gbp2, Gbp3, Gbp6, Gbp7, Ifitm3, Igtp, Irgm1, Irgm2, Stat1*, and *Tgtp1,* were involved in both pathways (Fig. 1E).

### *Gbp4* and *Irgb6* are expressed in various OB-resident and OB-infiltrating cells in response to IFN-γ, but not type I IFNs

**Fig 2:**
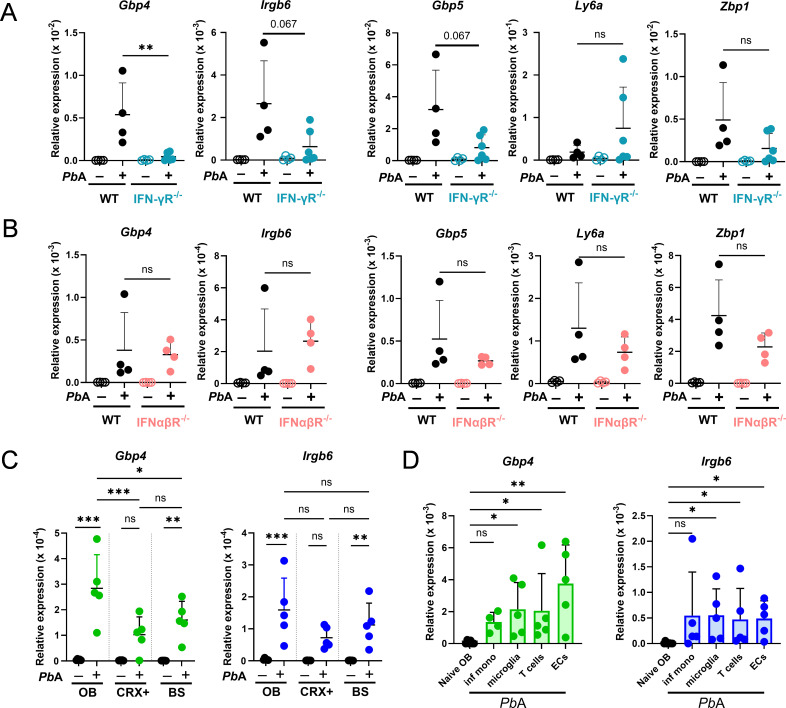
*Gbp4* and *Irgb6* are expressed in various OB-resident and OB-infiltrating cells in response to IFN-γ, but not type I IFNs. WT and *IFN-γR^−/−^* and *IFNα/βR^−/−^* mice were infected with *Pb*A, and OB were analyzed on days 0 and 6 post-infection. (**A and B**) Relative expressions of early expressed genes *Gbp4*, *Irgb6*, *Gbp5*, *Ly6a*, and *Zbp1* in OBs from WT and *IFN-γR^−/−^* mice (**A**), and *IFNα/βR^−/−^* mice on days 0 and 6 post-infection with *Pb*A parasites. (**C**) Relative expressions of *Gbp4* and *Irgb6* in OB, cortex, and brainstem of WT mice at days 0 and 6 post-infection with *Pb*A parasites. (**D**) RNAs were extracted from FACS-sorted infiltrating monocytes, T cells, microglia, and endothelial cells from the olfactory bulb of *Pb*A-infected mice, and the relative expression of *Gbp4* and *Irgb6* was measured. Data are shown as mean ± SD from *n* = 4–5 mice/group (**A through C**). Data are pooled from four independent experiments and shown as mean ± SD (pooled from eight mice, collected day 6, moribund) (**D**). Mann-Whitney test (**A, B**), one-way ANOVA with multiple comparisons test (**C**), and Kruskal-Wallis test (**D**) were used for the statistical analysis. *, *P* < 0.05; **, *P* < 0.01; ***, *P* < 0.001; ns, not significant.

To determine the inducibility of these genes by type I or type II IFNs, we collected OB from naïve and infected IFN-γ receptor knockout (*IFN-γR^−/−^*) and type I interferon receptor knockout (*IFNα/βR^−/−^*) mice and examined for their transcripts by quantitative PCR (qPCR) (Fig. 2A and B). The relative expression of *Gbp4* was significantly reduced (**P < 0.01), while *Irgb6* and *Gbp5* showed a trend toward reduction (*P* = 0.067), but no change was observed in *Ly6a* or *Zbp1* expression in infected *IFN-γR^−/−^* mice compared to wild-type (WT) controls (Fig. 2A). This suggests the importance of type II IFN signaling in the induction of these genes. Conversely, the relative expression levels of *Gbp4, Irgb6, Gbp5*, *Ly6a*, and *Zbp1* were similar between infected IFN-α*/β*R^−/−^ and WT counterparts, suggesting a lesser role for type I IFN in their induction (Fig. 2B).

Next, qPCR analysis was performed on three brain regions, OB, brainstem (BS), and the remainder of the brain (CRX+), from both naïve and *Pb*A-infected mice. This analysis revealed a significantly higher expression of *Gbp4* in the OB compared to the other regions following infection (Fig. 2C). The expression pattern of *Irgb6* in OB mirrored that of *Gbp4*. To identify the cellular sources of *Gbp4* and *Irgb6* expression in the *Pb*A-infected OB, we FACS-sorted infiltrating monocytes, microglia, T cells, and endothelial cells from the OB of day 6 *Pb*A-infected WT mice (Fig. S4) and extracted RNA for qPCR analysis. We found that both *Gbp4* and *Irgb6* were induced across all sorted populations, including brain-resident microglia and endothelial cells, as well as T cells and monocytes, the majority of which infiltrate the OB after *Pb*A infection (Fig. 2D). These findings suggest that both OB-resident and OB-infiltrating cells contribute to *Gbp4* and *Irgb6* expression during *Pb*A infection. Taken together, we identified *Gbp4* and *Irgb6* as the top two IFN-γ-inducible genes upregulated early during ECM and focused on further analysis of their roles in cellular immunity during ECM.

### Gbp4 and Irgb6 contribute to ECM development

**Fig 3:**
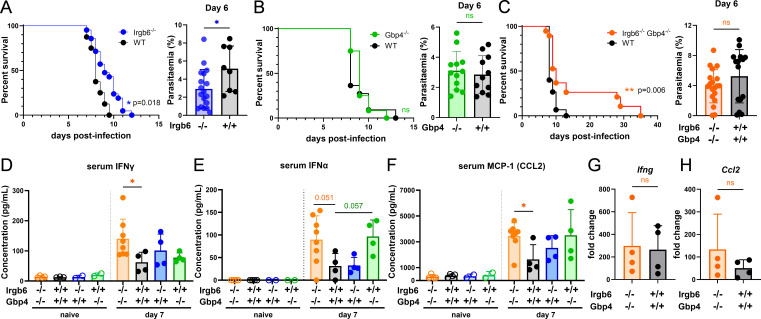
Gbp4 and Irgb6 contribute to ECM development. Groups of mice were infected with 10^5^ *Pb*A-GFP iRBCs. (**A**) Survival and day 6 parasitemia of *Irgb6^−/−^* (*n* = 21) and WT (*n* = 8) mice. (**B**) Survival and day 6 parasitemia of *Gbp4^−/−^* (*n* = 12) and WT (*n* = 11) mice. (**C**) Survival and day 6 parasitemia of *Irgb6^−/−^ Gbp4^−/−^* (*n* = 19) and WT (*n* = 15) mice. (**D through F**) Detection of IFN-γ (**D**), IFN-α (**E**), and MCP-1/Ccl2 (**F**) protein levels in the sera of *Irgb6*^−/−^, *Gbp4*^−/−^, *Irgb6*^−/−^ *Gbp4*^−/−^, and WT mice at days 0 and 7 post-infection (*n* = 4–8 mice/group) by either multiplex cytokine detection or enzyme-linked immunosorbent assay (ELISA). (**G and H**) Relative expressions of *Ifng* (**G**) and *Ccl2* (**H**) in the olfactory bulb of WT and *Irgb6^−/−^ Gbp4^−/−^* mice on day 6 post-infection with 10^6^ *Pb*A iRBCs relative to naïve (*n* = 4 mice/group). Survival comparisons between two groups were analyzed by the log-rank Mantel-Cox test (**A through C**). Data are shown as mean ± SD. Mann-Whitney test was used for statistical analysis. *, *P* < 0.05; **, *P* < 0.01; ns, not significant.

Next, to characterize the functions of *Gbp4* and *Irgb6 in vivo,* we used *Irgb6*-deficient (*Irgb6*^−/−^) mice (30) and newly generated *Gbp4*-deficient (*Gbp4*^−/−^) mice using CRISPR/Cas9 technology. Homozygous *Gbp4*^−/−^ mice were obtained after backcrossing and genotyping offspring heterozygous for the disrupted gene (Fig. S5). We infected *Gbp4*^−/−^, *Irgb6*^−/−^, and WT control mice with *Pb*A parasites and observed parasitemia and host survival (Fig. 3A and B; Fig. S6A and B). Significantly prolonged survival and reduced parasitemia were observed in the *Irgb6*^−/−^ mice compared to WT counterparts (Fig. 3A), but not in the *Gbp4*^−/−^ mice (Fig. 3B). The reduced parasitemia in the *Irgb6*^−/−^ mice may suggest potential direct or indirect involvement of this gene promoting the parasite’s growth in the periphery. GBPs have previously been shown to interact with Irgb6 (21, 35); thus, to seek whether Gbp4 and Irgb6 synergize as IFN-γ-inducible cell autonomous immunity molecules, we generated *Irgb6- Gbp4-* double-deficient (*Irgb6*^−/−^ *Gbp4*^−/−^) mice by crossing the *Irgb6*^−/−^ and *Gbp4*^−/−^ mice. Approximately 30% of *Pb*A-infected *Irgb6*^−/−^ *Gbp4*^−/−^ mice escaped ECM (**P = 0.006) with no difference in peripheral parasitemia compared to WT controls (Fig. 3C; Fig. S6C). Mice that escaped ECM in *Irgb6*^−/−^ *Gbp4*^−/−^ mice finally succumb to death due to high parasitemia and anemia (Fig. S6C and D).

Several cytokines are induced during ECM and have been associated with varying degrees of disease severity (36). To assess whether Gbp4 and Irgb6 influence the serum cytokine profiles during ECM, we measured serum cytokine levels using a multiplex assay (Fig. 3D through F; Fig. S7). Increased levels of IFN-γ and IFN-ɑ were detected in the serum of *Irgb6*^−/−^ *Gbp4*^−/−^ mice compared to WT at day 7 post-infection (Fig. 3D and E). Additionally, increased MCP-1 (CCL2) levels were measured in the serum of *Irgb6*^−/−^ *Gbp4*^−/−^ mice compared to WT on day 7 post-infection (Fig. 3F). No differences were observed in the other cytokines analyzed (IFN-β, IL-1α, IL-1β, IL-2, IL-3, IL-4, IL-5, IL-6, IL-9, IL-10, IL-12 (p40), IL-12 (p70), IL-13, IL-17A, Eotaxin, G-CSF, GM-CSF, KC, MIP-1α, MIP-1β, RANTES, TNF-α) ( Fig. S7). These findings may suggest a potential role of Gbp4 and Irgb6 in the negative regulation of the systemic production of IFN-ɑ, IFN-γ, and CCL2 during *Pb*A infection.

To determine whether systemic serum cytokine production reflects local events in the OB, we analyzed OB gene expression on day 7 post-infection. qPCR of the OB indicated no significant difference in *Ifng* nor *Ccl2* between *Irgb6*^−/−^ *Gbp4*^−/−^ mice and WT controls (Fig. 3G and H).

### Despite BBB disruption, olfaction is better preserved in *Irgb6*^−/−^ *Gbp4*^−/−^ mice

**Fig 4:**
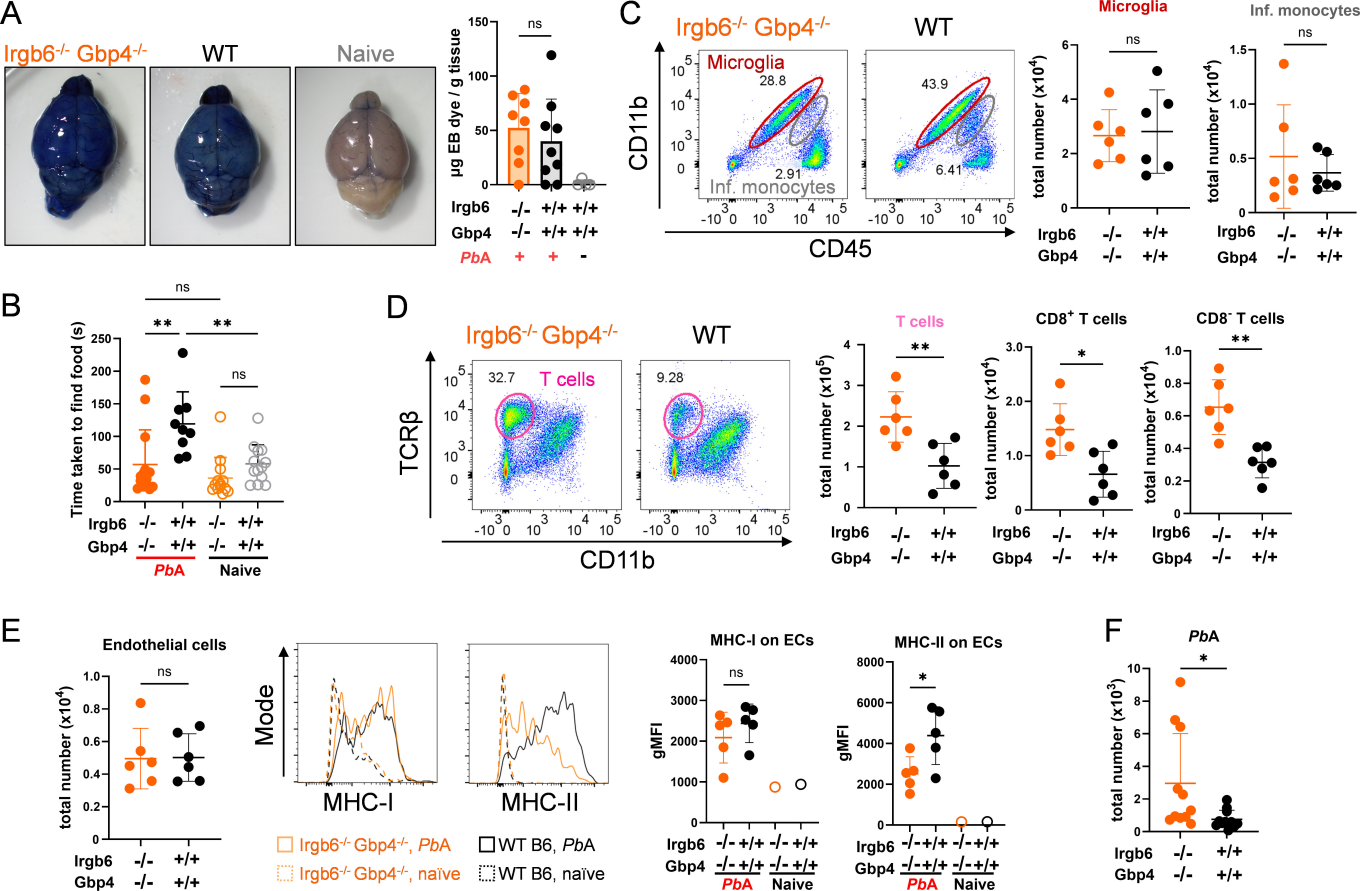
*In vivo* assessment of infected *Irgb6^−/−^ Gbp4^−/−^* mice during ECM. Groups of *Irgb6^−/−^ Gbp4^−/−^* and WT mice were infected for the *in vivo* assessment of ECM. (**A**) Representative images of Evans blue dye injected mice from naïve and infected groups and Evans blue dye quantification in OB of *Pb*A-infected WT, *Irgb6^−/−^ Gbp4^−/−^*, and naïve WT mice on day 8 post-infection (*n* = 4–8 mice/group). (**B**) Buried food test of naïve and *Pb*A-infected WT and *Irgb6^−/−^ Gbp4^−/−^* mice on day 7 post-infection. Time taken to find hidden food is shown in seconds (*n* = 9–13 mice/group). (**C and D**) Representative flow cytometry plots and absolute cell numbers of infiltrating monocytes and microglia (**C**), and TCRβ^+^, CD8^+^ T, and CD8^−^ T cells (**D**) in the olfactory bulb of WT and *Irgb6^−/−^ Gbp4^−/−^* mice at day 8 post-infection. Each data point represents a sample pooled from 2 to 3 mice, normalized by the number of mice used (*n* = 6 mice/group). (**E**) Absolute cell counts of endothelial cells and their MHC-I and -II expression are shown as geometric mean fluorescence intensity (gMFI), in the olfactory bulb of WT and *Irgb6^−/−^ Gbp4^−/−^* mice at day 8 post-infection. (**F**) Quantified numbers of GFP-*Pb*A in the olfactory bulb of WT and *Irgb6^−/−^ Gbp4^−/−^* mice at day 8 post-infection. Each data point represents an OB randomly pooled from 2 to 3 mice in each group and normalized by the number of mice. Scatter plots present the mean ± SD. Data were analyzed by using one-way ANOVA with multiple comparisons test (**B**), Student’s t test (**A, C through E**), and Mann-Whitney test (**F**). *, *P* < 0.05; **, *P* < 0.01; ns, not significant.

To understand the reasons for improved survival in the *Irgb6*^−/−^ *Gbp4*^−/−^ mice, the ECM disease phenotype was further investigated. Evans Blue (EB) dye, which is known for its high affinity for serum albumin, was injected intravenously into *Pb*A-infected mice 1 h before sampling to assess BBB integrity. EB dye staining of the brain on day 8 post-infection was comparable between *Irgb6*^−/−^ *Gbp4*^−/−^ and WT mice, indicating similar levels of BBB disruption (Fig. 4A). However, buried food test scores (13), measuring the mice’s ability to smell, were significantly better in *Pb*A-infected Irgb6^−/−^ *Gbp4*^−/−^ mice compared to WT, correlating with their improved survival (Fig. 4B). Although our transcriptomic analysis in WT mice revealed significant downregulation of GPCR signaling pathway genes, including the olfactory receptor (OR) genes, and genes related to olfactory bulb development after *Pb*A infection (Fig. S3A through C), olfaction in the *Irgb6*^−/−^ *Gbp4*^−/−^ mice remained unaffected. Together, these data may suggest the involvement of IFN-inducible GTPases in olfaction.

### Increased T cell recruitment in the OB of *Pb*A-infected *Irgb6*^−/−^ *Gbp4*^−/−^ mice

We next analyzed resident and infiltrating immune cell populations in the OB on days 7 or 8 post-infection. There was no difference in the numbers and MHC-I and MHC-II expressions of resident microglia and infiltrating monocytes (37) between *Irgb6*^−/−^ *Gbp4*^−/−^ and WT mice (Fig. 4C; Fig. S8 and S9). Interestingly, *Irgb6*^−/−^ *Gbp4*^−/−^ mice exhibited a significant increase in T cell populations, including both CD8^+^ and CD8^−^ T cells, compared to WT (Fig. 4D). Notably, although there was no difference in the number of CD45^−^ CD31^+^ endothelial cells between the groups, *Irgb6*^−/−^ *Gbp4*^−/−^ mice exhibited markedly reduced MHC-II activation, but not MHC-I, in endothelial cells following *Pb*A infection (Fig. 4E). Additionally, we observed an increase in accumulated *Pb*A parasite counts in the OB of *Irgb6*^−/−^ *Gbp4*^−/−^ mice compared to WT by flow cytometry, which was confirmed by CUBIC (clear, unobstructed, brain/body imaging cocktails and computational analysis)-cleared whole brain imaging (Fig. 4F; Fig. S10). Taken together, these findings suggest that while *Irgb6*^−/−^ *Gbp4*^−/−^ mice exhibit similar BBB disruption to WT, they display significantly better preservation of olfaction, increased T cell and *Pb*A parasite accumulation, and decreased MHC-II activation on ECs.

### Recruited CD4^+^ and CD8^+^ T cells in the OBs of *Irgb6*^−/−^ *Gbp4*^−/−^ mice display reduced functionality

**Fig 5:**
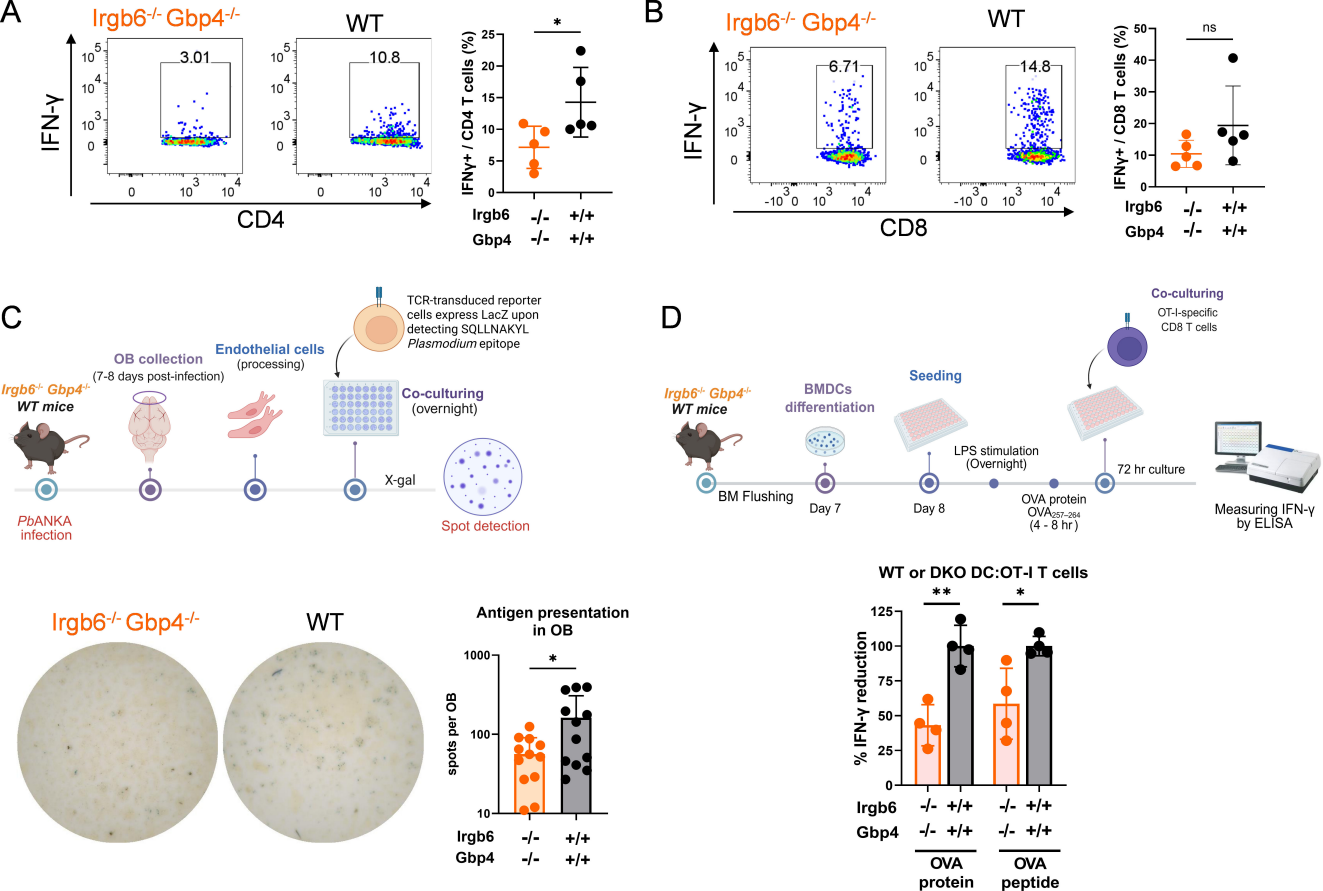
Despite higher brain infiltrating CD4^+^ and CD8^+^ T cells, less cross-presentation results in less IFN-γ production in *Irgb6^−/−^ Gbp4^−/−^* mice OBs. (**A and B**) Flow cytometry analysis of IFN-γ^+^ CD4 T cells (**A**) and IFN-γ^+^ CD8 T cells (**B**) in the olfactory bulb of WT and *Irgb6^−/−^ Gbp4^−/−^* mice on day 8 after *Pb*A infection. Each dot represents a sample pooled from 2 to 4 mice. Representative flow cytometry plots are shown. (**C**) Schematic diagram of the experimental protocol and the results of *ex vivo* endothelial cell antigen cross-presentation assay from *Pb*A-infected WT and *Irgb6^−/−^ Gbp4^−/−^* mice in the OB. Representative wells are shown. Data were pooled from three independent experiments, *n* = 12 for each group. Each dot represents a sample from one mouse. (**D**) Schematic diagram of the experimental protocol and IFN-γ results of BMDC: OT-I CD8 T cell co-culture assay. LPS-activated BMDCs from WT and *Irgb6^−/−^ Gbp4^−/−^* mice were induced with OVA or OVA_257–264_ peptide prior to co-culture with purified OT-I CD8^+^ T cells for 3 days at a 1:1 ratio. Concentrations of IFN-γ in the culture supernatants were measured by ELISA. Data were depicted as %IFN-γ-reduction compared to WT mice values and pooled from the two independent experiments, *n* = 4 for each group. Each dot represents a sample from one mouse. Data are shown as mean ± SD and analyzed with Student’s t test. *, *P* < 0.05; **, *P* < 0.01; ns, not significant.

We questioned whether the increased T cell population in *Irgb6*^−/−^ *Gbp4*^−/−^ mice is functional, given that their improved survival was counterintuitive to the established pathogenic role of T cells, particularly CD8^+^ T cells, during ECM. To investigate this, we measured IFN-γ production by brain-infiltrating T cells (Fig. 5A and B; Fig. S11A for gating strategy). We found a significantly lower proportion of IFN-γ^+^ CD4^+^ T cells (*P < 0.05), and a trend toward less proportion of IFN-γ^+^ CD8^+^ T cells, in the OB of *Irgb6*^−/−^ *Gbp4*^−/−^ mice compared to WT mice, suggesting impaired IFN-γ production in these T cells. Consistent with the findings in the OB, we also observed significantly lower proportions of IFN-γ^+^ CD4^+^ T cells (**P < 0.001) and IFN-γ^+^ CD8^+^ T cells (*P < 0.05) in other brain regions (Fig. S11B and C), indicating that the recruitment of less functional T cells occurs throughout the brain. Taken together, our data suggest the limited functionality of the OB- and brain-recruited T cells in the *Irgb6*^−/−^ *Gbp4*^−/−^ mice, despite their higher infiltration.

### Antigen presentation perturbed in *Irgb6*^−/−^ *Gbp4*^−/−^ mice

An important aspect of T cell recruitment to the brain during ECM involves the cross-presentation of parasite antigens by brain endothelial cells (38, 39). We hypothesized that the reduced functionality of OB-infiltrating T cells in *Irgb6*^−/−^ *Gbp4*^−/−^ mice might result from impaired antigen presentation. To test this, we performed an *ex vivo* antigen cross-presentation assay (Fig. 5C). We utilized the NFAT-lacZ reporter T cell line LR-BSL8.4a, which expresses a TCR specific for the Pb1 epitope (SQLLNAKYL, derived from the GAP50 protein) presented by mouse H-2D^b^ MHC class I molecules. Upon recognition of the antigen, these reporter T cells express β-galactosidase and stain blue following X-gal treatment (39). These reporter cells were co-incubated with brain microvessels isolated from *Pb*A-infected mice at day 7/8 post-infection when they exhibited ECM symptoms, stained with X-gal, and the resulting blue spots were counted. We observed a significant decrease in antigen presentation in the OB of *Irgb6^−/−^ Gbp4^−/−^* mice compared to WT counterparts (Fig. 5C).

Next, we investigated whether *Irgb6^−/−^ Gbp4^−/−^* mice exhibit a reduced capacity to present other antigens other than Pb1, such as OVA, *in vitro*. Bone marrow-derived DCs (BMDCs) isolated from WT and *Irgb6^−/−^ Gbp4^−/−^* mice were pulsed with either OVA protein or the OVA_257–264_ peptide and co-cultured with OT-I CD8^+^ T cells to assess IFN-γ production by the T cells (40). IFN-γ production by OT-I cells co-cultured with *Irgb6^−/−^ Gbp4^−/−^* BMDCs was significantly reduced (by more than 40%) compared to those co-cultured with WT BMDCs (Fig. 5D). Furthermore, there was no difference between stimulation with OVA protein versus OVA peptide, indicating that *Irgb6* and *Gbp4* are broadly involved in both antigen processing and presentation, during ECM and potentially in other contexts.

## DISCUSSION

**Fig 6:**
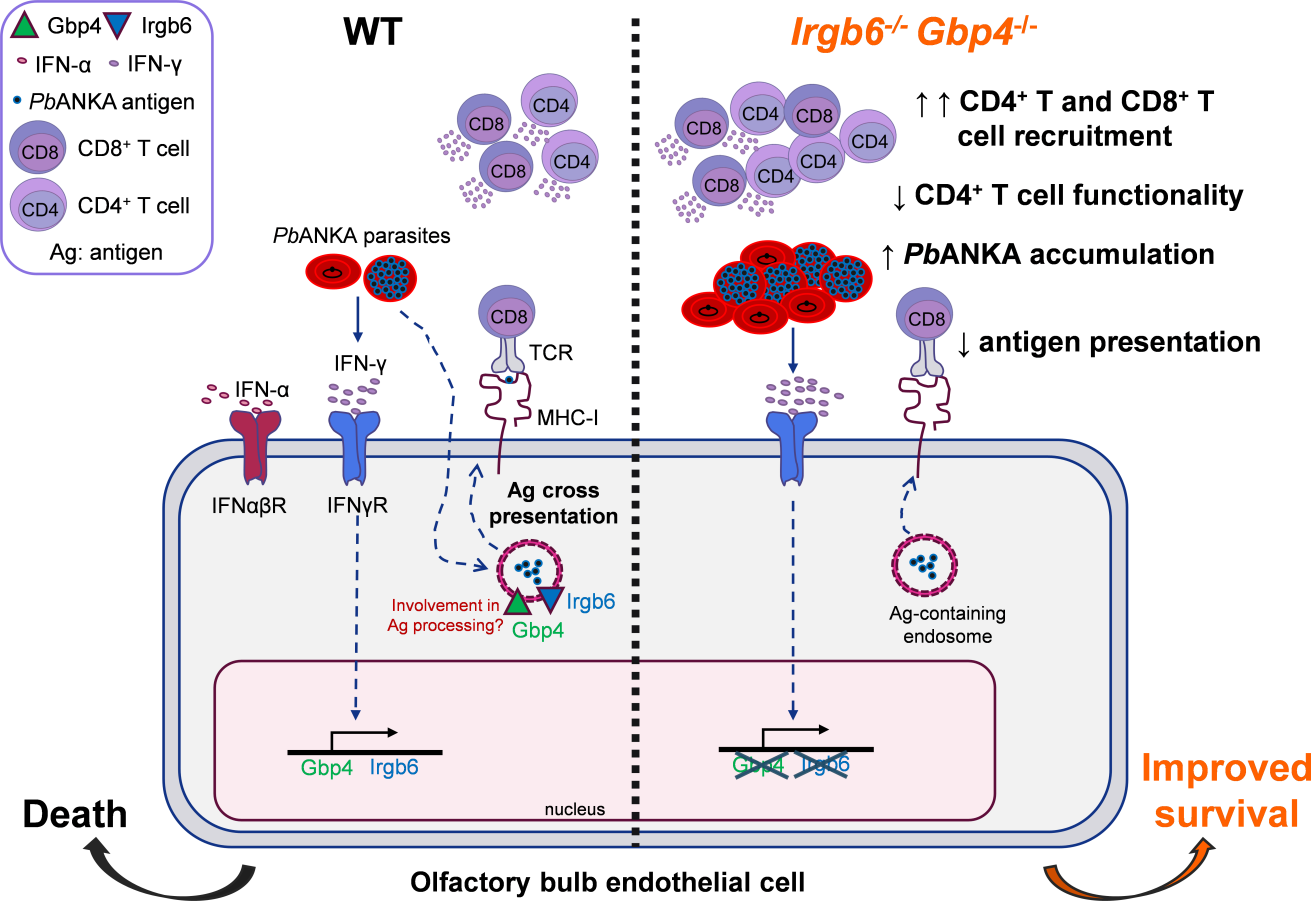
Proposed model for the role of GTPases Gbp4 and Irgb6 during experimental cerebral malaria in the OB. In WT mice, infection with *Pb*A triggers systemic production of IFN-γ, which binds to IFN-γ receptors on endothelial cells in the OB and induces the expression of Gbp4 and Irgb6. These endothelial cells then process and cross-present *Pb*A antigens to infiltrating T cells, leading to the ECM pathology. In *Irgb6^−/−^ Gbp4^−/−^* mice, the absence of these GTPases results in increased *Pb*A accumulation, increased CD4^+^ and CD8^+^ T cell infiltration, but impaired T cell functionality. Additionally, the disruption of *Pb*A antigen cross-presentation in these double-knockout mice suggests that Gbp4 and Irgb6 play roles in antigen processing and presentation. These findings overall indicate that Gbp4 and Irgb6 are key components in regulating immune responses in the OB, while helping to coordinate effective pathogen control, which in turn leads to excessive immune activation and pathology.

The OB has emerged as a particularly vulnerable region in the brain during ECM, which exhibits earlier and more extensive BBB disruption and a higher density of microhemorrhages compared to other brain areas. In this study, we investigated the contribution of the OB to early ECM pathogenesis using transcriptomic profiling. Our analysis revealed a marked early upregulation of ISGs, notably the interferon-inducible GTPases *Gbp4* and *Irgb6*, in the OB of *Pb*A-infected C57BL/6 mice. These genes were predominantly induced by IFN-γ and expressed in infiltrating monocytes, microglia, endothelial cells, and T cells. Functional studies demonstrated that *Gbp4* deficiency alone did not alter parasitemia or survival, whereas *Irgb6*-deficient mice displayed reduced parasitemia and improved survival outcomes. Notably, mice deficient in both *Gbp4* and *Irgb6* showed significantly enhanced survival despite unaltered peripheral parasitemia and BBB integrity. Further analyses revealed shifts in brain-infiltrating T cell populations and altered antigen presentation by OB endothelial cells in *Irgb6*^−/−^ *Gbp4*^−/−^ mice, suggesting that these GTPases modulate antigen presentation pathways and subsequent T cell responses during ECM (Fig. 6).

Although the critical role of IFN-γ in the induction of Gbp4 and Irgb6 in defence against various pathogens has been increasingly recognized, their specific contributions to ECM remained unclear, despite the well-established central role of IFN-γ in ECM pathogenesis (37, 41). Increased expression of *Gbp4* has been associated with several diseases, including cancer, leishmaniasis, chikungunya, and malaria (41–44). However, the function of Gbp4 remains poorly characterized, with the exception of its reported role in Sendai virus infection, where it interacts with IRF7 to act as a negative regulator of type I IFN production in macrophages (24). In contrast, the function of Irgb6 is better understood, particularly in the context of *T. gondii* infection, where it is upregulated in macrophages, localizes to the parasitophorous vacuole membrane, and restricts parasite replication (21, 30). In our study, we found that the upregulation of *Gbp4* and *Irgb6* during *Pb*A infection is dependent on type II IFN signaling. Interestingly*, Irgb6^−/−^* mice exhibited reduced parasitemia, suggesting a potential role for *Irgb6* in promoting parasite growth, which warrants further investigation. In contrast, *Gbp4^−/−^* mice displayed no significant phenotypic changes. Notably, the prolonged survival observed in *Irgb6^−/−^ Gbp4^−/−^* mice, despite unchanged parasitemia, suggests that these genes may act in concert to modulate immune responses during ECM.

Despite prolonged survival, *Irgb6*^−/−^ *Gbp4*^−/−^ mice exhibited elevated serum levels of IFN-γ, IFN-ɑ, and CCL2. CCL2, a key chemoattractant for monocytes and T cells, is known to drive immune cell infiltration into the brain during ECM and other neuroinflammatory conditions (36, 45–50). Notably, *Gbp4^−/−^* deficient mice alone showed increased serum IFN-α, consistent with Gbp4’s reported role in negatively regulating IFN-α via IRF7 during viral infection (24). This elevation likely drives CCL2 upregulation, which in turn may influence BBB permeability (51). However, Evans blue dye analysis revealed that BBB disruption in *Irgb6*^−/−^ *Gbp4*^−/−^ mice remained comparable to WT controls, and OB *Ccl2* expression was unchanged. These findings suggest that while systemic CCL2 may contribute to BBB alterations, additional factors—both local and systemic—likely modulate BBB integrity and survival outcomes in these mice.

Although peripheral parasitemia did not differ between *Irgb6*^−/−^ *Gbp4*^−/−^ mice and WT mice, an increased accumulation of *Pb*A parasites was observed in the OB, as detected by flow cytometry and confirmed with CUBIC-cleared brain images. This was accompanied by a significant increase in infiltrating CD4^+^ and CD8^+^ T cells in the OB, along with a decrease in MHC-II expression by OB endothelial cells. One potential explanation for the increased T cell infiltration in the OB of *Irgb6*^−/−^ *Gbp4*^−/−^ mice compared to WT mice could be the elevated parasite load in the gene-deficient mice. The increased parasite burden may enhance endothelial cell activation, which in turn contributes to the observed pathology (52). Another possibility is that, despite peripheral parasitemia being comparable to WT mice, the *Irgb6*^−/−^ *Gbp4*^−/−^ mice may have higher parasitemia overall, but due to sequestration of parasites in the OB, this difference is masked in peripheral blood measurements.

Alternatively, the increased parasite accumulation in the brain of *Irgb6*^−/−^ *Gbp4*^−/−^ mice may be driven by increased T cell infiltration. Previous studies have suggested the critical role of parasite accumulation in the brain for the development of ECM (53, 54). Both CD4^+^ and CD8^+^ T cells have been implicated in promoting parasite accumulation in the brain and other vital organs, such as lungs, with IFN-γ having a direct role in this process (55, 56). In a study using *CD8^−/−^* mice and anti-CD8 treatment, a reduction in parasite biomass and iRBC accumulation in the brain and spleen was observed (56). Furthermore, perforin-deficient *C57BL/6 prf^−/−^* mice showed diminished iRBC accumulation in the brain (57), suggesting a possible role of perforin in brain parasite accumulation. These previous studies altogether may help to explain the increased parasite accumulation observed in the brains of *Irgb6*^−/−^ *Gbp4*^−/−^ mice in our study.

During ECM, IFN-γ activates brain endothelial cells, upregulates MHC-I expression (58), and promotes cross-presentation of parasite antigens. A hallmark of ECM pathology is the infiltration of cytotoxic CD8^+^ T cells into the brain, where antigen presentation by endothelial cells is essential for disease development (38, 39). Interestingly, *Irgb6*^−/−^ *Gbp4*^−/−^ mice, which exhibit prolonged survival, display increased T cell infiltration in the OB, a seemingly paradoxical finding. This could reflect either enhanced infiltration from peripheral organs or local T cell proliferation. However, splenic T cell numbers were similar between *Irgb6*^−/−^ *Gbp4*^−/−^ and WT mice (data not shown), and although serum CCL2 was elevated in *Irgb6*^−/−^ *Gbp4*^−/−^ mice, OB expression of its receptor, CCR2, remained unchanged (data not shown). Given that IFN-γ^+^ CD4^+^ T cells promote pathogenic CD8^+^ T cell accumulation during ECM (59), their reduction in *Irgb6*^−/−^ *Gbp4*^−/−^ mice may contribute to disease attenuation. These mice showed impaired T cell functionality, with reduced frequencies of IFN-γ^+^ CD4^+^ and CD8^+^ T cells in the brain. Nonetheless, absolute numbers of these cytokine-producing T cells were similar to WT, possibly explaining why a subset (~70%) of *Irgb6*^−/−^ *Gbp4*^−/−^ mice still succumb to ECM.

Our data further demonstrate that endothelial cells isolated from the OB of *Pb*A-infected *Irgb6*^−/−^ *Gbp4*^−/−^ mice exhibit reduced antigen cross-presentation compared to those isolated from infected WT mice. While these findings suggest that MHC-II-mediated antigen presentation may play a more prominent role than MHC-I in the observed phenotype of *Irgb6*^−/−^ *Gbp4*^−/−^ mice, a suitable model to directly assess MHC-II-dependent antigen presentation during malaria is currently lacking. Therefore, we evaluated the cross-presentation capacity of OB endothelial cells within the context of MHC-I. The outcomes of this assay may reflect differences in cross-presentation efficiency, local antigen availability, and/or the rate of endothelial cell death (60). Considering the established roles of GBPs and IRGs in the targeting and disrupting pathogens and pathogen-containing vacuoles (reviewed in reference 61), we speculate that Gbp4 and Irgb6 might be involved in the initial steps of antigen processing, which has been shown to occur through the phagosome-to-cytosol route, by destabilizing parasites or parasite-containing vacuoles (12) within the cell and making the antigen available for further processing. As studies have shown the recruitment of IRGs and GBPs to phagosomes (21), it is plausible that Gbp4 and Irgb6 could also be involved in processing *Plasmodium* antigens.

We utilized a full knockout of *Gbp4* and *Irgb6,* rather than a cell-type-specific conditional knockout; thus, the observed T cell phenotype could originate either from T cell-intrinsic mechanisms or from other cells, such as antigen-presenting cells that interact with them. Since Irgb6 (also known as Tgtp1/2, T-cell-specific guanine nucleotide triphosphate-binding protein 1 and 2) was originally identified as a protein predominantly expressed in T cells (27), further investigation into its specific role in T cells may be worthwhile. However, considering the established role of IFN-inducible GTPases in host immune defence, especially against toxoplasma and bacterial infections (20, 61), we suspect that Gbp4 and Irgb6 may have a more significant impact on APCs, including endothelial cells. Moreover, studies using IFN-γR2 conditional knockout mice have demonstrated that T-cell intrinsic IFN-γ signaling does not directly drive pathogenic T cell responses during ECM. Instead, IFN-γ modulates brain chemokine levels and promotes CD8^+^ T cell recruitment (58). As Gbp4 and Irgb6 are both IFN-γ inducible, we speculate that their roles in ECM and the phenotype we observed may primarily involve non-T cells. An open question remains as to whether Gbp4 and/or Irgb6 influence IFN-γ signaling. Nevertheless, the observation under non-malarial conditions that IFN-γ production by OT-I cells co-cultured with *Irgb6^−/−^ Gbp4^−/−^* BMDCs was significantly reduced compared to those co-cultured with WT BMDCs may suggest that *Irgb6* and *Gbp4* are broadly involved in both antigen processing and presentation.

In addition to *Gbp4* and *Irgb6*, other genes were also upregulated by day 3 post*-Pb*A infection. Among the most highly upregulated was *Ly6a* (lymphocyte activation protein-6A), also known as *Sca-1* (stem cell antigen-1). Ly6a/Sca-1 is a glycosylphosphatidylinositol-anchored surface molecule expressed by various immune cell populations and commonly used as a marker for hematopoietic stem cells (62, 63). *In vitro* studies have shown that both type I IFN and IFN-γ strongly induce Sca-1 expression in naïve CD4^+^ and CD8^+^ T cells via STAT1 signaling (64). In addition, previous studies have demonstrated Sca-1 expression in brain-infiltrating macrophages (65) and CD31^+^ endothelial cells during ECM (58). Given that IFN-γ induces Sca-1 expression on hematopoietic and non-hematopoietic cells, we speculate that IFN-γ similarly induces Sca-1 expression in the OB of *Pb*A-infected mice. *Gvin1* was also significantly upregulated on day 3 post-*Pb*A infection. Gvin1 (GTPase, very large interferon inducible 1) belongs to the very large inducible GTPase (VLIG) family, another group of IFN-inducible GTPases (66). Previous studies have reported *Gvin1* upregulation in the brain of *Pb*A-infected mice (67); however, its specific role in the pathology of ECM remains unclear.

In mice, IRGs are classified into regulatory (e.g., Irgm1, Irgm2, and Irgm3), effector (e.g., Irga6, Irgb6, Irgb10, and Irgd), and decoy proteins (68). Regulatory IRGs localize to host organelle membranes such as mitochondria, ER, and Golgi, and inhibit effector IRGs under homeostatic conditions. In pathogen-containing vacuoles lacking regulatory IRGs, membranes are marked for effector IRG recruitment (reviewed in reference 68). *Irgb6* localization is regulated by *Irgm1* and *Irgm3*, as reduced *Irga6* and *Irgb6* expression is observed in *Irgm1*- and *Irgm3*-deficient mice (29, 69). Notably, *Irgm3^−/−^* mice are protected from ECM, displaying reduced IFN-γ and CCL2 levels, and impaired accumulation of CD4^+^ and CD8^+^ T cells in the brain (70). Although the mechanism remains unclear, impaired antigen presentation may be involved, as *Irgm3* has been implicated in cross-presentation (71). Similarly, *Irgm1^−/−^* mice exhibit elevated type I IFN and CCL2, consistent with features of type I interferonopathy and autoimmunity (72, 73). These findings raise the possibility that *Gbp4* and *Irgb6* may be compensated by other IRGs and may also contribute to autoimmune processes.

Olfactory dysfunction is a common feature of several diseases, including neurodegenerative disorders such as Alzheimer’s disease and respiratory infections such as COVID-19 (74, 75). We have also previously demonstrated a loss of smell function in mice during ECM (13). Currently, no studies directly link GBPs or IRGs to olfactory function. The reduced decline in olfaction observed in *Pb*A-infected *Irgb6^−/−^ Gbp4*^−/−^ mice could be due to better preservation of neural tissues in the OB, which are typically damaged during ECM, as olfactory dysfunction could be caused by damage/loss of the olfactory epithelium or the olfactory processing pathways of the central nervous system (76). However, since BBB disruption was comparable between *Irgb6^−/−^ Gbp4*^−/−^ mice and their WT counterparts, Gbp4 and Irgb6 are unlikely to play a role in maintaining the BBB integrity. Nonetheless, the mechanism by which the loss of Irgb6 and Gbp4 improves olfactory function remains unclear and needs further investigation.

We also observed a differential expression of genes related to olfactory receptor (OR) activity at late ECM in the WT mice. ORs are GPCRs typically activated by volatile chemical compounds in the nasal olfactory epithelium and transfer the signal to the primary olfactory cortex. Decreased OR expression at late ECM in the WT could be reflective of the disrupted olfaction observed in those mice. However, recent studies have revealed that the function of ORs goes beyond sensing odorants in the olfactory epithelium and have implicated ORs in glucose metabolism, inflammatory disease, and cancer (reviewed in reference 77). OR expression has been observed in non-neuronal cell types, including immune cells such as macrophages, where they are involved in the activation of NLR family pyrin domain containing 3 (NLRP3) inflammasome and induction of interleukin-1β secretion (78). Furthermore, OR gene expression has also been observed in human malaria patients’ whole blood samples (79), and a recent study in mice described the role of Olfr1386 in regulating type I interferon (IFN-I) responses during malaria parasite infections (80). Thus, the increased expression of some of these ORs might be reflective of their involvement in immune response against the *Pb*A parasites, beyond their known roles in olfaction.

In summary, our findings emphasize the roles of Gbp4 and Irgb6 in modulating immune responses during ECM, particularly in the OB. Their influence on T cell functionality and antigen presentation offers new insights into the mechanisms of ECM pathogenesis and potential therapeutic targets for mitigating cerebral malaria.

## MATERIALS AND METHODS

### Mice

Wild-type C57BL/6J (WT) mice were purchased from CLEA Japan (Tokyo, Japan). B6.129S7-Ifngr1tm1Agt/J (IFN-γR^−/−^) mice were purchased from The Jackson Laboratory (JAX stock #003288) (81). IFNα/βR^−/−^ (A129) mice were purchased from B&K Universal and backcrossed to B6 for more than eight generations as previously described (82). Irgb6^−/−^ C57BL/6 mice (30) were kindly provided by Professor M. Yamamoto (Osaka University, Osaka, Japan).

*Gbp4^−/−^* C57BL/6 mice were generated using the CRISPR/Cas9 technology as previously described (83). Two single guide RNAs, 5′-CATCACAAGTGATGAGTACC-3′ and 5′-CCAATTGGATCCTACGTTTA-3′, designed to recognize the coding region of the *Gbp4* gene at the fifth exon, were purchased from IDT (Coralville, IA, USA). Primer sequences used for *Gbp4* genotyping were F-5′-CCTTCTGGGATCTGAGTCACC-3′ and R-5′-ACCACCACCAACAACAACAAACTC-3′ (Fig. S3).

### *Plasmodium berghe*i ANKA infection

Mice were inoculated intraperitoneally with 10^6^ or 10^5^ *Pb*A-infected erythrocytes GFP-labeled (*Pb*A-GFP) or not (WT-*Pb*A), kindly provided by Prof. Yuda, Mie University (13). Peripheral blood parasitemia was evaluated daily by Giemsa-stained thin blood smears from day 6 post-infection onward.

### RNA isolation, sequencing, and analysis

OBs of mice from naïve, day-3, and day-6 post-infection with WT-*Pb*A were sampled into TRIzol reagent (Invitrogen) after transcardial perfusion with 30 mL of ice-cold D-PBS. RNAs were purified according to the manufacturer’s instructions (Invitrogen). The total RNA integrity was confirmed using the RNA 6000 Nano kit on an Agilent 2100 Bioanalyzer, ensuring all samples had an RIN ≥8. A cDNA library was constructed using the TruSeq stranded mRNA library prep kit (Illumina). RNA sequencing was performed by the Genome Information Research Center of Osaka University using the Illumina HiSeq 2500 platform in 75-base single-end mode. Sequenced reads were mapped to the mouse reference genome sequence GRCm38 (mm10) using TopHat2, BowTie2, and SAMtools. Mapped sequenced reads were assigned to genomic features, including counts, using Rsubread featureCounts. Counts were normalized using DESeq2 via SARTools (84) for differential expression analysis, and the Benjamini-Hochberg procedure was applied to control the FDR at a level of 0.05. One sample from the day 3 group was excluded from further analysis as an outlier for low intra-group correlation after quality control analysis with iDEP (32). Genes were considered as differentially expressed when they had |log_2_ fold-change| ≥ 1.0 and adjusted *P*-value < 0.05. This widely used criterion (31) is equivalent to a twofold change, which captures biologically meaningful changes without being too stringent to balance sensitivity and specificity. Volcano plots and heatmaps were generated using the EnhancedVolcano and ComplexHeatmap packages in R. The clusterProfiler (85) and GSEA software version 4.3.3 (86) were used for GO enrichment analysis, and Cytoscape software v. 3.10.2 was used for the visualization (87).

### Reverse transcription quantitative PCR

Brain tissues from the OB, cerebral cortex (CRX+), and BS were sampled into TRIzol from *Pb*A-GFP-infected mice, 6 days post-infection, and their naive controls after transcardial perfusion with ice-cold D-PBS. The tissue samples were homogenized, the total RNA extracted, and the cDNA prepared using ReverTra Ace (Toyobo) as previously described (13). Quantitative PCR was performed using THUNDERBIRD Probe qPCR Mix (Toyobo) and Taqman primers for *Ccl2*, *Gbp4*, *Gbp5*, *Ifng*, *Ly6a*, *PbANKA* 18s rRNA, *Tgtp1 (Irgb6*), and *Zbp1* (Applied Biosystems). Gene expressions were normalized to the expression level of eukaryotic 18S rRNA endogenous control.

### CUBIC tissue-clearing and light sheet fluorescent microscope imaging

To perform CUBIC clearing of the brain, reagents and whole brain tissues were processed as previously described (14). Briefly, mice were anesthetized and transcardially perfused with PBS, followed by 4% paraformaldehyde (PFA). The brains were shaken in CUBIC-L for delipidation, stained with Alexa Fluor 594-conjugated anti-α-smooth muscle actin (α-SMA, Abcam; ab202368) antibody at 1:50 dilution, then immersed in CUBIC-R for refractive-index matching before microscopy.

The CUBIC-treated and antibody-stained brain samples were immersed in an oil mixture (RI = 1.525) and imaged using a custom-built light-sheet fluorescent microscope (LSFM, MVX10-LS, Olympus). A 0.63× objective lens with 488 and 594 nm emission lasers was used to acquire the images. The acquired images were volume rendered and analyzed using Imaris software.

### Isolation of brain leukocytes

Brain leukocytes were isolated using a modified method described previously (13). Briefly, after transcardial perfusion with ice-cold D-PBS, mice brains were sliced up into a digestion solution containing RPMI 1640 with L-Gln (Nacalai tesque), 10% heat-inactivated fetal bovine serum (Sigma Aldrich), 750 U/mL deoxyribonuclease I (Wako Fujifilm), and 200 U/mL collagenase (Wako Fujifilm), and they were incubated at 37°C in a water bath with shaking for 30 min. Then, cells were filtered through a 70 µm cell strainer, centrifuged at 1,500 × *g* rpm for 10 min at 4°C, and pelleted. The cell pellets were resuspended in 30% Percoll and layered over a 70% Percoll gradient and centrifuged at 1,300 × *g* for 30 min at 4°C. The leukocytes were collected at the interphase and washed with D-PBS before counting and staining for flow cytometry.

### Flow cytometry

Brain-isolated leukocytes were incubated with Fc blocker (BioLegend) for 5 min at room temperature before staining with fluorophore-conjugated surface-stain antibody mixtures at 4°C for 25 min. Dead cells were excluded using the LIVE/DEAD fixable dead cell stain (Invitrogen). Anti-CD11b (M1/70), CD8 (53-6.7), CD31 (390), CD45 (30-F11), H-2Kb/H-2Db (28-8-6), Ly6C (HK1.4), and TCRβ (H57-597) antibodies were purchased from BioLegend, and I-A/I-E (M5/11.15.2) was purchased from eBioscience. Data acquisition was performed using the LSRFortessa (BD Biosciences) flow cytometer, and sorting was performed by using BD Influx (BD Biosciences) (88). Data were analyzed with FlowJo 10.10.0.

### Intracellular cytokine staining

Isolated brain leukocytes were cultured in 200 µL RPMI 1640 with L-Gln (Nacalai Tesque) supplemented with 10% FBS and 1% penicillin/streptomycin (Nacalai Tesque). The cells were cultured in the presence of BD GolgiPlug Protein Transport Inhibitor containing Brefeldin A (BD Bioscience) for 6 h, after which they were stained with LIVE/DEAD fixable dead cell stain (Invitrogen), washed, then incubated with Fc blocker and subsequently with anti-CD11b (M1/70), CD4 (RM4-5), CD8 (53-6.7), CD45 (30-F11), and TCRβ (H57-597) antibodies purchased from Biolegend. These surface-stained cells were fixed with 1% PFA (Nacalai Tesque) in PBS for 20 min, permeabilized with BD Perm/Wash buffer (BD Biosciences), and intracellularly stained with anti-mouse IFN-γ (XMG1.2, Biolegend). Sample events were acquired using an LSRFortessa flow cytometer (BD Biosciences) and the data analyzed with Flowjo 10.10.0.

### Serum cytokines

Sera were collected from *Pb*A-GFP-infected mice on days 0 and 7 post-infection for cytokines and chemokines detection by Bio-Plex Pro Mouse cytokine 23-plex (Bio-Rad). IFN-α and IFN-β were quantified using a mouse IFN-α enzyme-linked immunosorbent assay (ELISA) kit (PBL Assay Science) and mouse IFN-β DuoSet ELISA (R&D Systems), respectively.

### Assessment of BBB permeability

To assess BBB integrity, WT mice were followed for the onset of ECM symptoms, as mice typically become moribund around day 7 or 8 post-infection. Mice were injected intravenously with 200 µL of 2% Evans blue (EB) dye (Sigma Aldrich) in PBS. One hour after EB dye injection, mice were anesthetized and transcardially perfused. The brains were imaged using a stereo microscope (Olympus SZX7) and weighed. The OB was separated from the brain and submerged in formamide (Nacalai Tesque) for 24 h, and the optical density (OD) of the extracted dye was measured at 640 nm. The OD was used to calculate the concentration of the dye per gram of OB tissue.

### Buried food test

The buried food test was performed as previously described (13). Briefly, mice were left without food overnight on day 6, one day prior to the anticipated onset of symptoms, and were placed in a new cage the next day containing food buried under the bedding. The time taken for mice to find the buried food was recorded in seconds.

### Endothelial cell *ex vivo* presentation assay

Brain microvessels were isolated from *Pb*A-infected mice at 7–8 days post-infection, when WT mice showed ECM symptoms as described previously (39). Briefly, OB was collected after terminal exsanguination of the mice, sliced, then passed through a 23-gauge needle. The homogenized tissue was gradient-centrifuged with 30% wt/vol dextran (Sigma), and the brain microvessel fragments were collected on a 40 µm cell strainer. These fragments were digested with 1 mg/mL collagenase IV (Gibco) and 10 µg/mL DNase (Roche) in D-PBS (Nacalai Tesque) containing 2% FBS for 1.5 h at room temperature. The digested fragments were washed, resuspended in RPMI complete media, and added to a well containing 3 × 10^4^ cells of the cloned TCR-transduced cell line LR-BSL8.4a (that recognizes the *Pb*A epitope SQLLNAKYL and induces *LacZ* expression), kindly provided by Prof. L. Renia, in a 96-well filter plate. After overnight co-incubation, the wells were stained with X-gal and images captured using an ImmunoSpot Analyzer S6 Ultimate M2 (Cellular Technology Limited). The spots were counted using ImageJ and reported per OB.

### Generation of bone marrow-derived DC

BMDC generation was performed as previously described (40). Bone marrow cells were flushed from the femurs and tibias of WT and *Irgb6^−/−^ Gbp4^−/−^* mice with RPMI 1640 medium containing 10% FBS using a 23G needle. Cells were subsequently filtered using a 70 µm cell strainer, centrifuged, and treated with ACK lysis buffer for red blood cell lysis. After washing in PBS, cells were resuspended in BMDC differentiation medium, which contained RPMI 1640 medium supplemented with 10% FBS and 100 ng/mL recombinant Flt3 ligand (PeproTech; Cat# 250-31L) at a concentration of 4 × 10^6^ cells/mL and incubated for 8 days. On day 8, differentiated BMDCs were collected and used in co-culture experiments.

### BMDC: OT-1 CD8 T cell co-culture assay

*In vitro* OT-I CD8 T cell activation assay was conducted as previously reported, with modifications (40, 89). BMDCs derived from WT and *Irgb6^−/−^ Gbp4^−/−^* mice were seeded on 96-well U-bottom plates at 5 × 10^4^ cells/well concentration and treated overnight with 100 ng/mL LPS. Cells were subsequently primed with 0.5 mg/mL OVA (Kanto Chemical, Japan) and 0.1 nM OVA_257–264_ peptide (H-2K^b^-restricted OVA class I epitope, MBL; TS5001-P) for 4 and 8 h, respectively. Following incubation, the unprocessed peptide was removed by washing the cells three times with PBS. OT-I CD8^+^ T cells were isolated from the spleens of OT-I mice (The Jackson Laboratory, Bar Harbor, ME) and enriched using MojoSort Mouse CD8 T Cell Isolation Kit (BioLegend; Cat# 480008). OT-I CD8+ T cells were then co-cultured with peptide-loaded BMDCs at a 1:1 ratio for 72 h. Following the co-culture time, culture supernatants were collected, and IFN-γ levels were measured by mouse IFN-γ DuoSet ELISA (R&D Systems) according to the manufacturer’s instructions.

### Statistical analysis

Graphs were plotted and analyzed with GraphPad Prism 10 software. The Mann-Whitney test or the Student’s t test was used for comparing two groups, while one-way analysis of variance (ANOVA) or the Kruskal-Wallis test was used for comparing more than two groups, with multiple comparisons. Survival between the two groups was compared by the log-rank Mantel-Cox test. Statistical significances are represented as ns, not significant; *P < 0.05, **P < 0.01, and ***P < 0.001. Some supplemental figures were created using BioRender.com.

## Data Availability

The RNA sequencing data have been deposited into NCBI’s Gene Expression Omnibus (GEO) database under accession no. GSE282591.

## SUPPLEMENTAL MATERIAL

Supplemental Figures mbio.01249-25-s0001.pdf Figures S1 to S11.

Supplemental Tables mbio.01249-25-s0002.xlsx Tables S1 and S2.

ASM does not own the copyrights to Supplemental Material that may be linked to, or accessed through, an article. The authors have granted ASM a non-exclusive, world-wide license to publish the Supplemental Material files. Please contact the corresponding author directly for reuse.
