# Supplementary material for: IFNγ-inducible Gbp4 and Irgb6 contribute to experimental cerebral malaria pathology in the olfactory bulb
Source: mBio. 2025 Jul 3;16(8):e01249-25. doi: 10.1128/mbio.01249-25 (PMC12345229; doi:10.1128/mbio.01249-25)
Supplement: Supplemental Figures — Figures S1 to S11. [file mbio.01249-25-s0001.pdf]

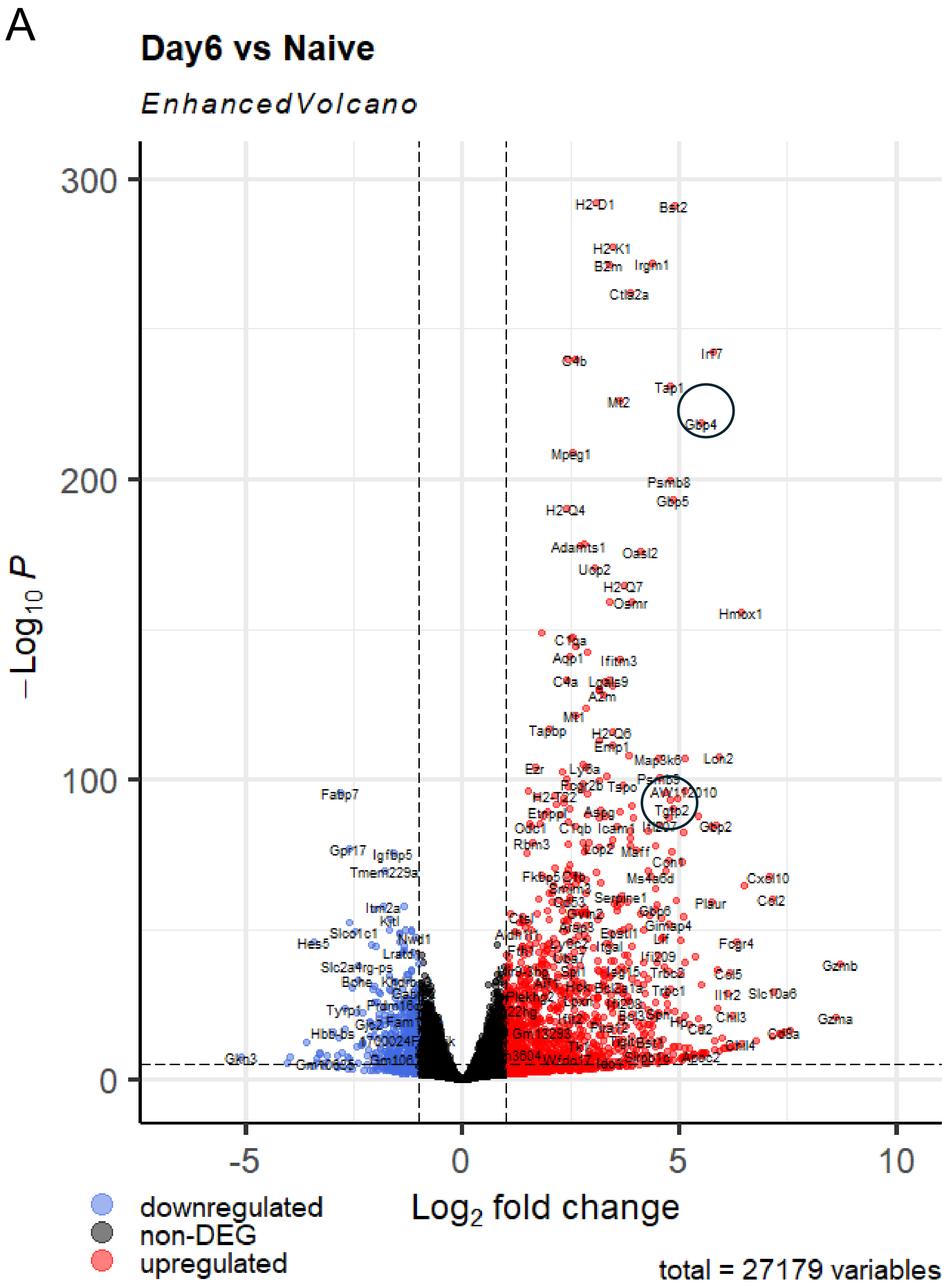

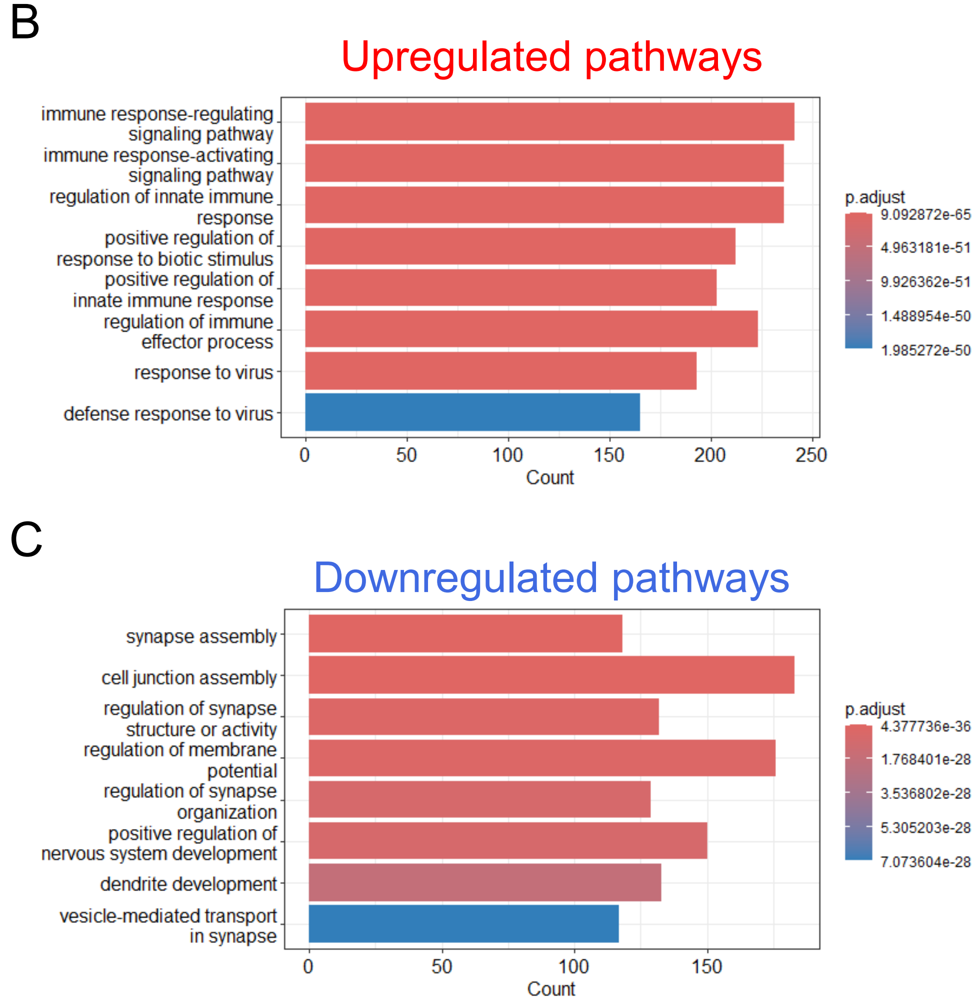

Supplementary Figure 1. Transcriptomic analysis of olfactory bulb (OB) at day 6 vs day 0 post-infection. (A) Volcano plot with cut-off $p \leq 0.05$, $|\log2 \text{ fold change}| \geq 1$. **(B)** Day 6 upregulated pathways, over representation analysis (GO biological process). **(C)** Day 6 downregulated pathways, over representation analysis (GO biological process).

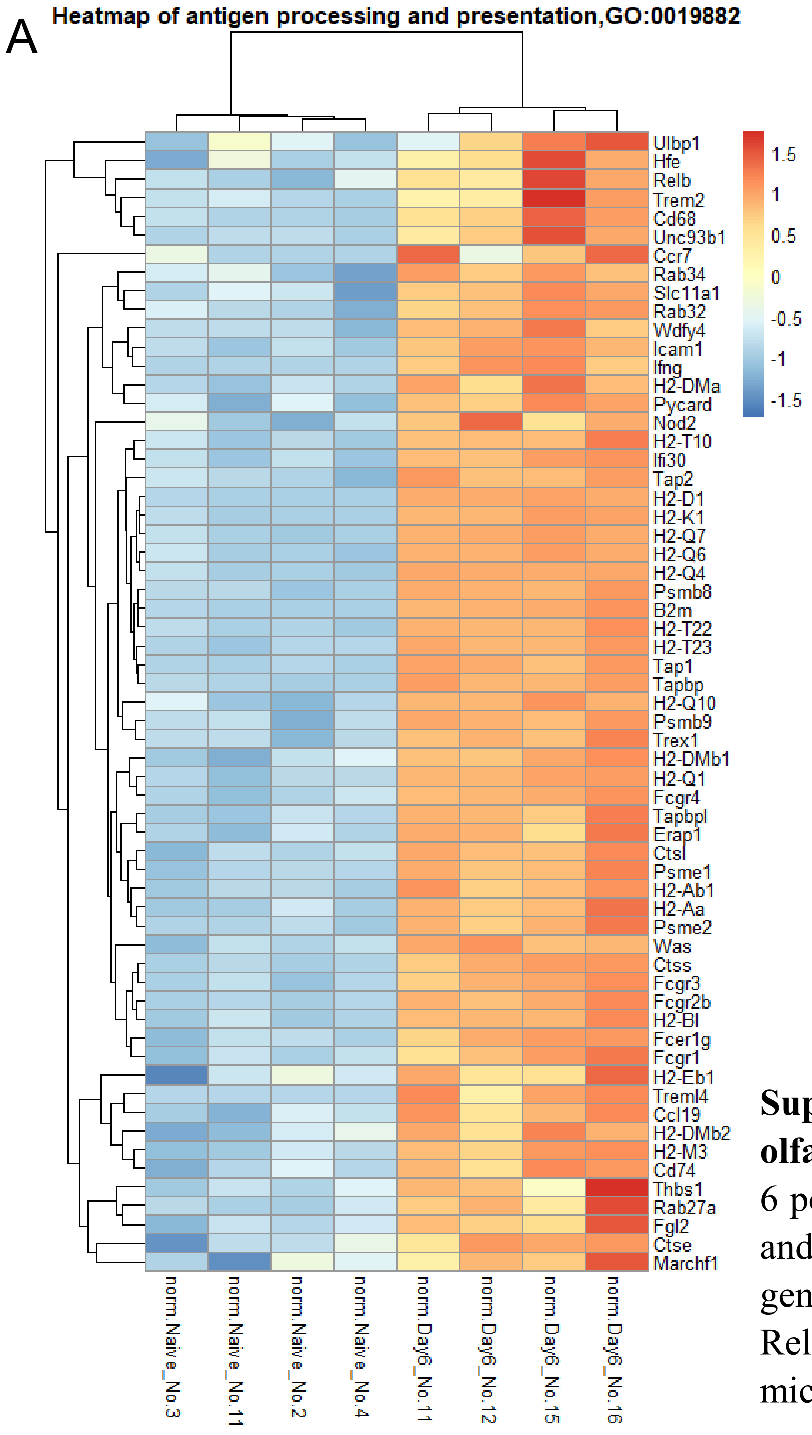

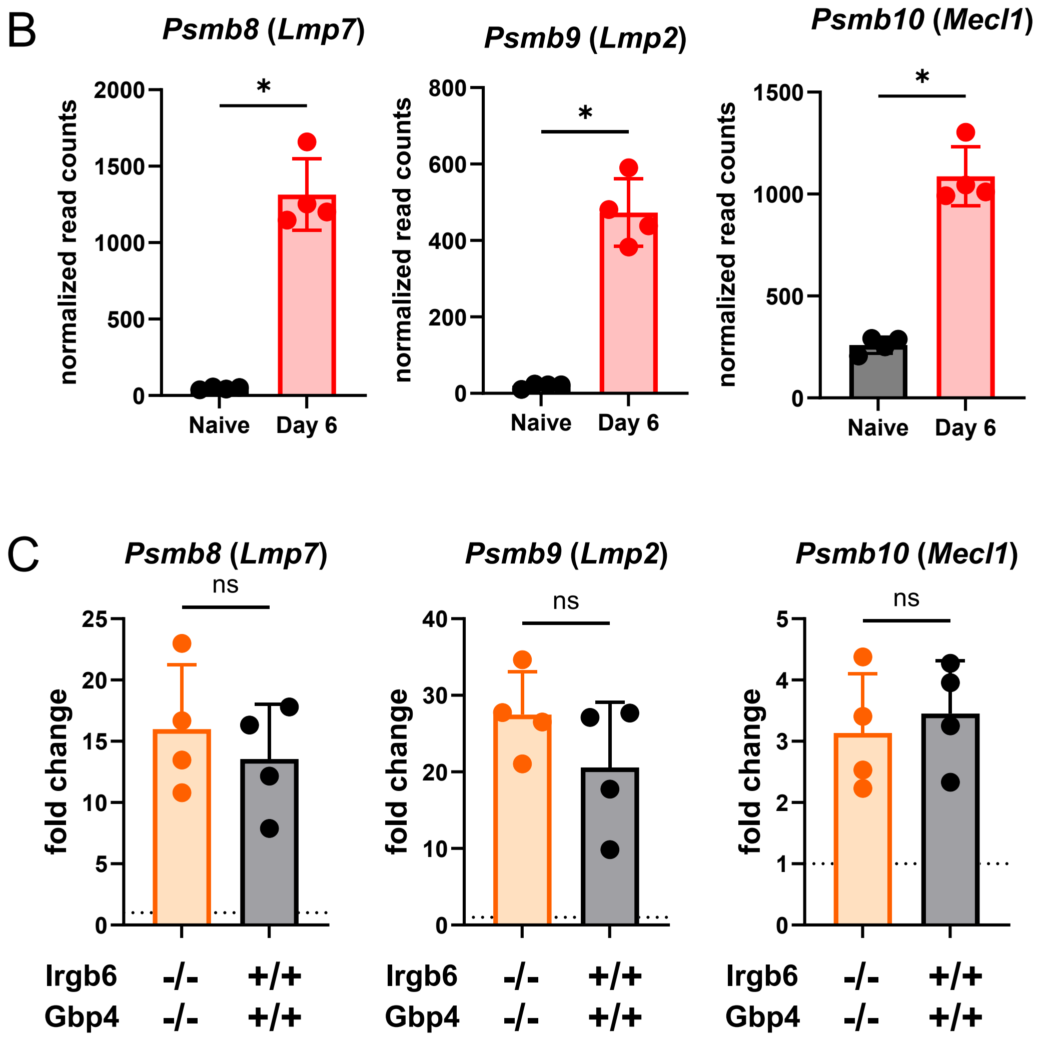

Supplementary Figure 2. Genes that encoding proteasome components in olfactory bulb (OB) on Day 6. (A) Heatmap of differentially expressed genes on day 6 post-infection ($p \leq 0.05$) categorized under gene ontology term “antigen processing and presentation”, GO:0019882. **(B)** Normalized read counts of immunoproteasome genes, *Psmb8, Psmb9,* and *Psmb10* in OB of naïve and day 6 *Pb*A post-infection. **(C)** Relative expressions of *Psmb8, Psmb9,* and *Psmb10* in OB of WT and *Irgb6*$^{-/-}$ *Gbp4*$^{-/-}$ mice on day 6 post-infection relative to naïve (n = 4 mice/group, ns: not significant).

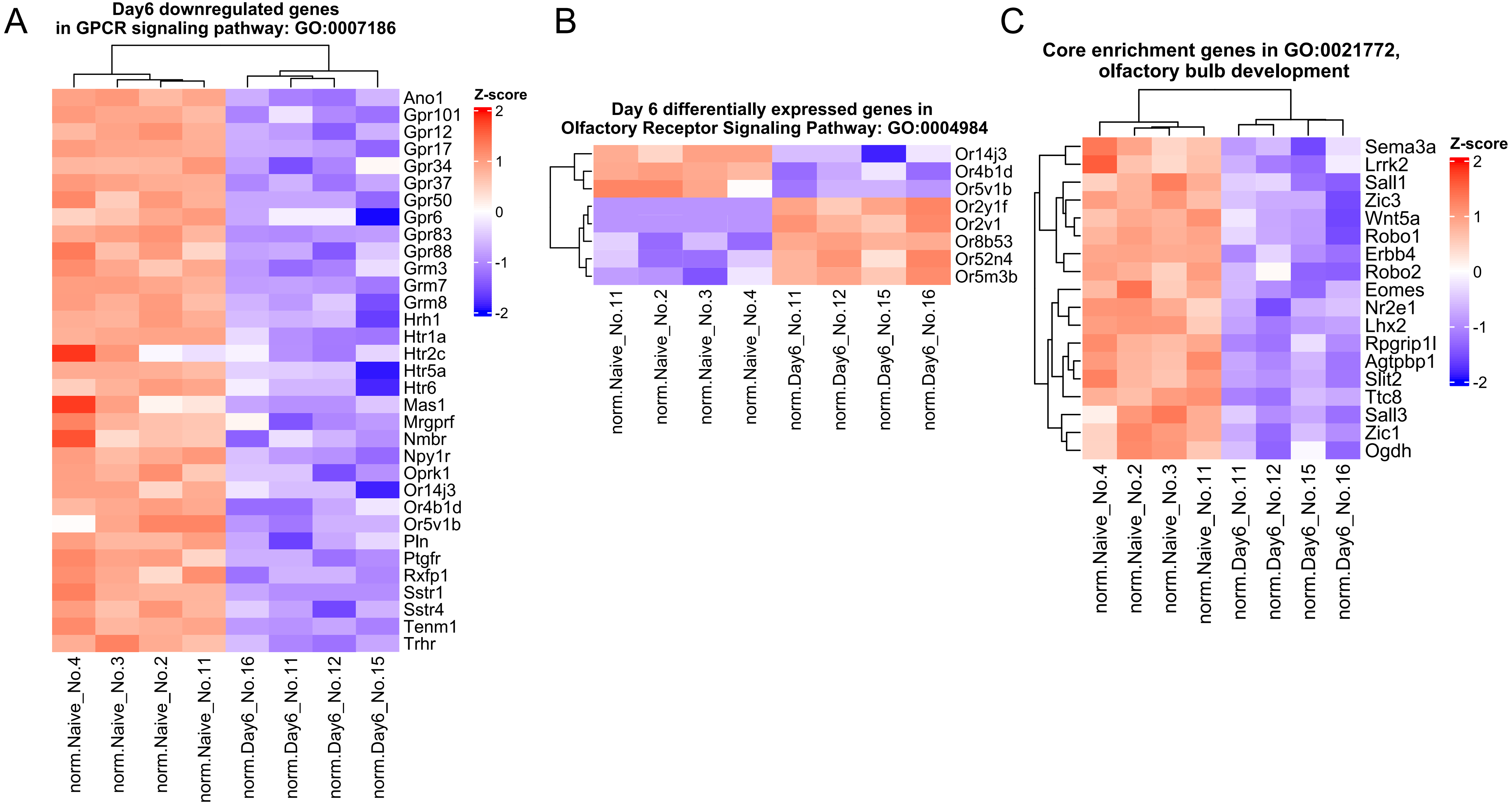

Supplementary Figure 3. Gene expression heatmaps of day 6 post-infection gene set for OB-related receptor and development genes. **(A)** Heatmap of differentially expressed genes downregulated on day 6 post-infection ($p \leq 0.05$, log2 fold change ≤ -1) categorized under the gene ontology term “G protein-coupled receptor signaling pathway”, GO:0007186. **(B)** Heatmap of differentially expressed genes on day 6 post-infection ($p \leq 0.05$, log2 fold change ≤ -1) categorized under gene ontology term “Olfactory receptor signaling pathway”, GO:0004984. **(C)** Heatmap of core enrichment genes in gene ontology term GO:0021772, olfactory bulb development, that were significantly enriched in the Day 6 post-infection gene set compared to Naïve.

Gating strategy for FACS sorting of OB infiltrating monocytes, T cells, microglia, and endothelial cells

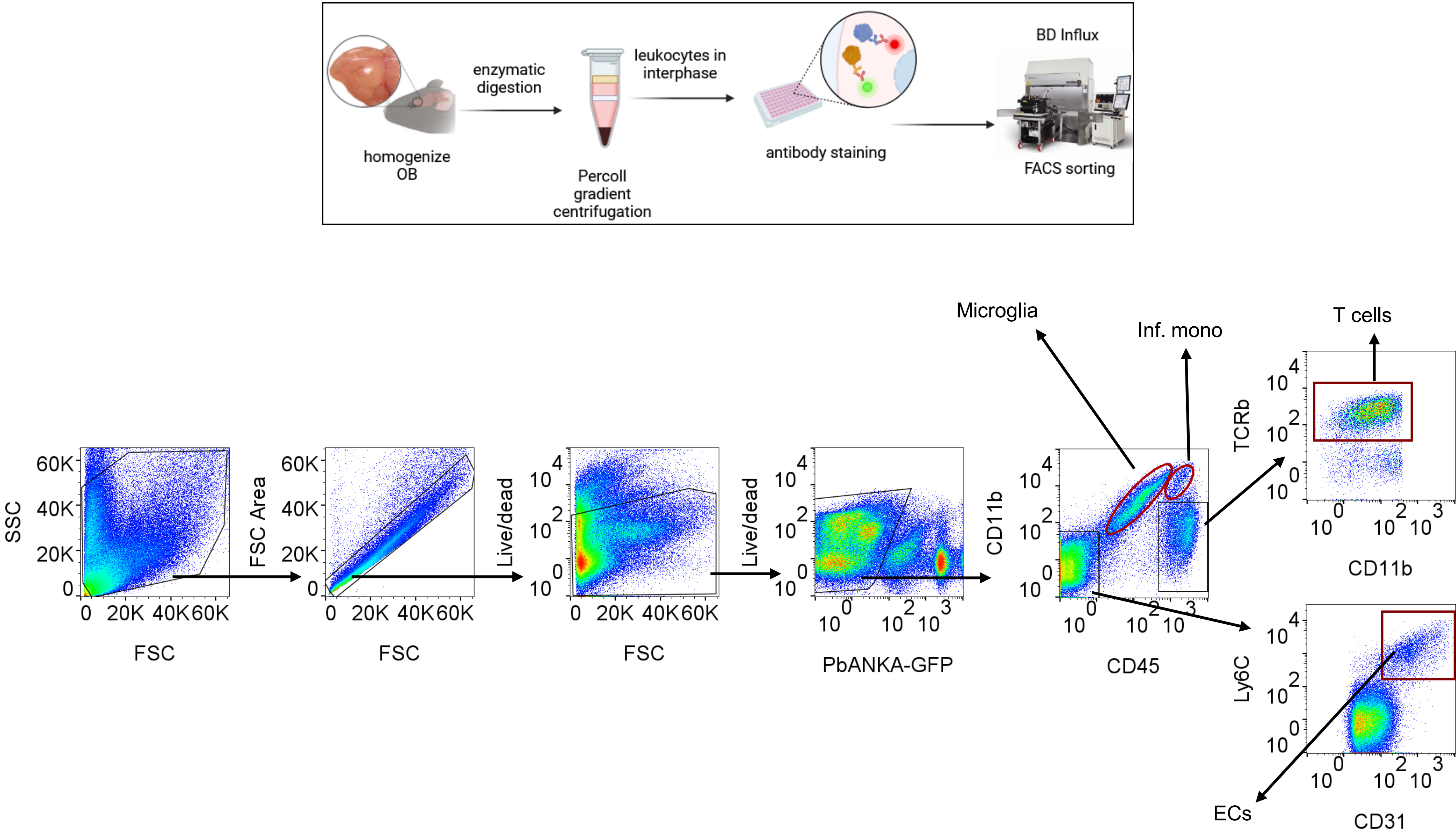

Supplementary Figure 4. Flow cytometry gating strategy for FACS sorting of OB infiltrating monocytes, T cells, microglia, and endothelial cells for data shown in Fig. 2.

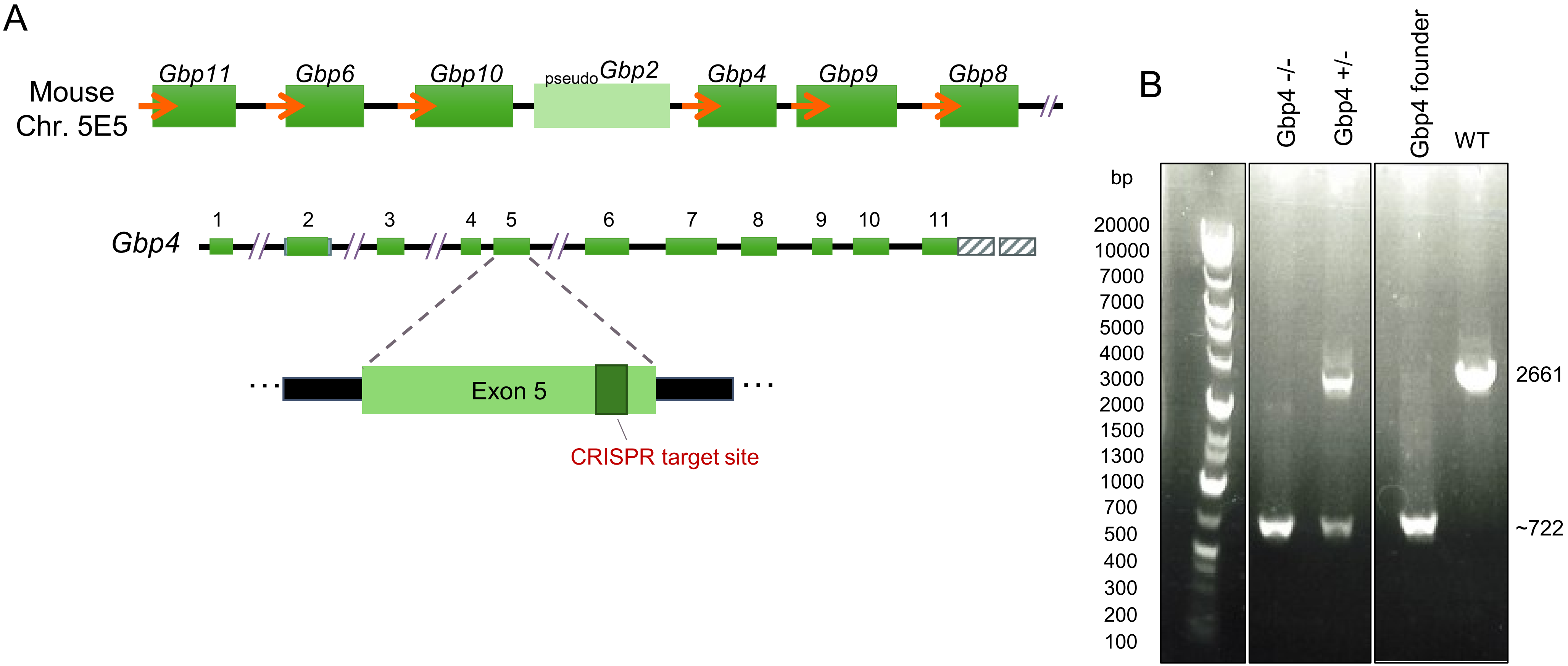

Genotyping PCR primers

Gbp4_genotype F	CCTTCTGGGATCTGAGTCACC
Gbp4_genotype R2	ACCACCACCAACAACAACAAACTC

Supplementary Figure 5. Generation of Gbp4 KO mouse. (A) Schematic representation of the gene-targeting strategy of Gbp4 locus by CRISPR-Cas9. **(B)** Genotyping of CRISPR-generated Gbp4 KO mouse.

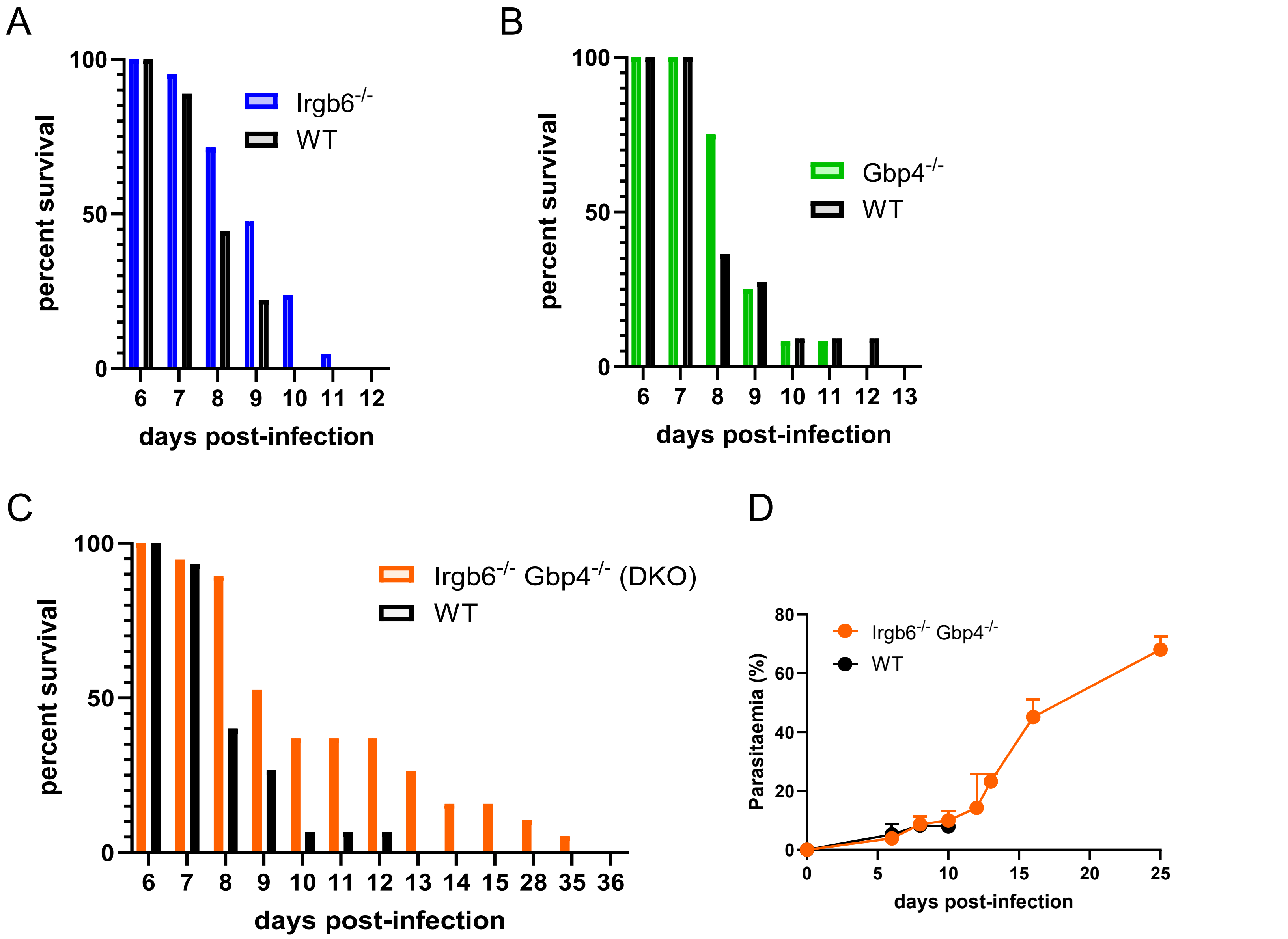

Supplementary Figure 6. Daily mice survival that experienced ECM at each timepoint after *Pb*A infection (related to Figure 3). Daily percent survival of Irgb6 $^{-/-}$ **(A),** Gbp4 $^{-/-}$ **(B),** and percent survival and parasitemia course of Irgb6 $^{-/-}$ Gbp4 $^{-/-}$ mice, respectively **(C and D).**

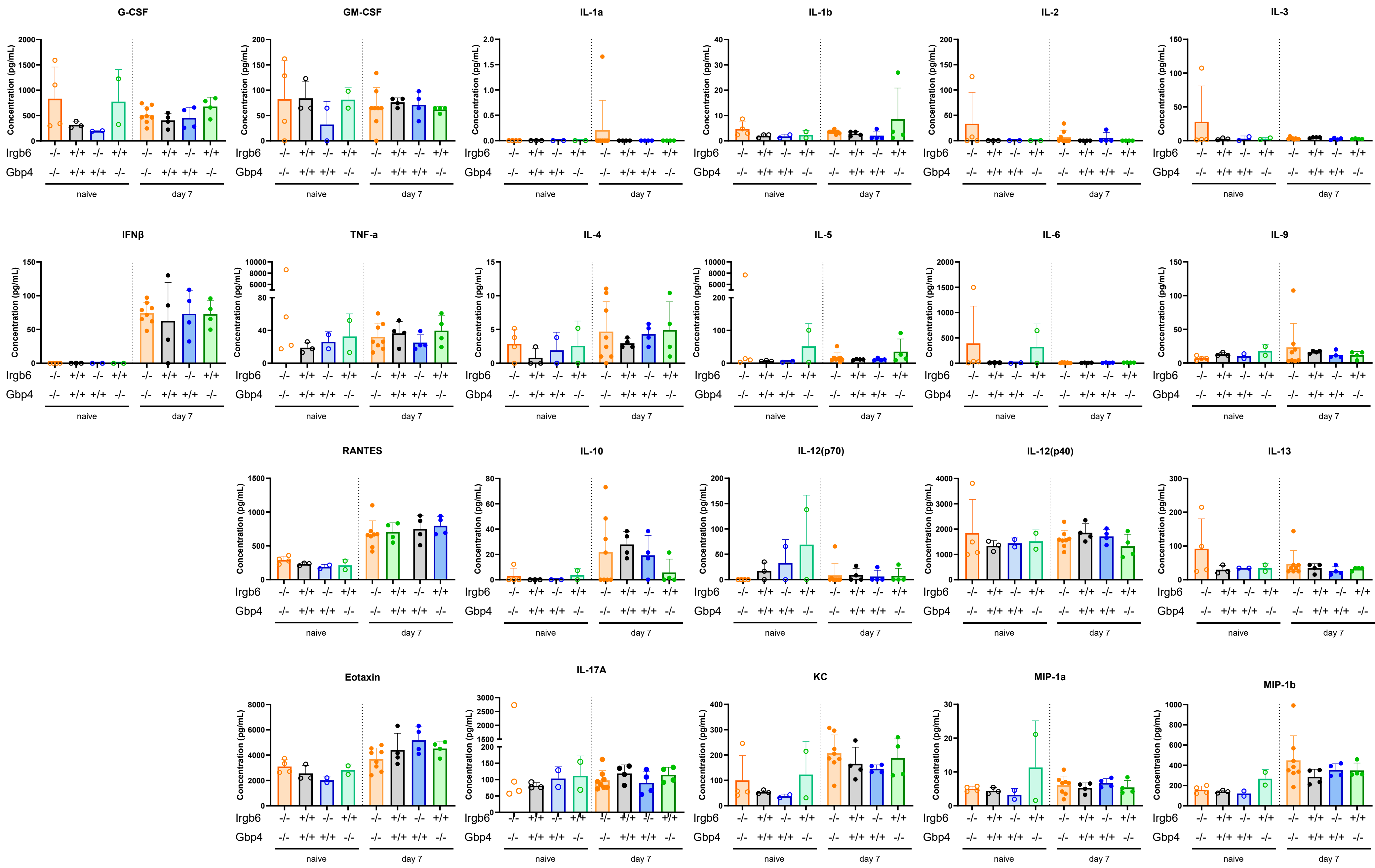

Supplementary Figure 7. ELISA (for IFN-β) and Bioplex multiplexing assay (for all others) of serum cytokines in in Irgb6 $^{-/-}$, Gbp4 $^{-/-}$, and Irgb6 $^{-/-}$ Gbp4 $^{-/-}$ mice day 7 post-infection, related to Figure 3.

Gating strategy for analysis of OB-infiltrating immune cells

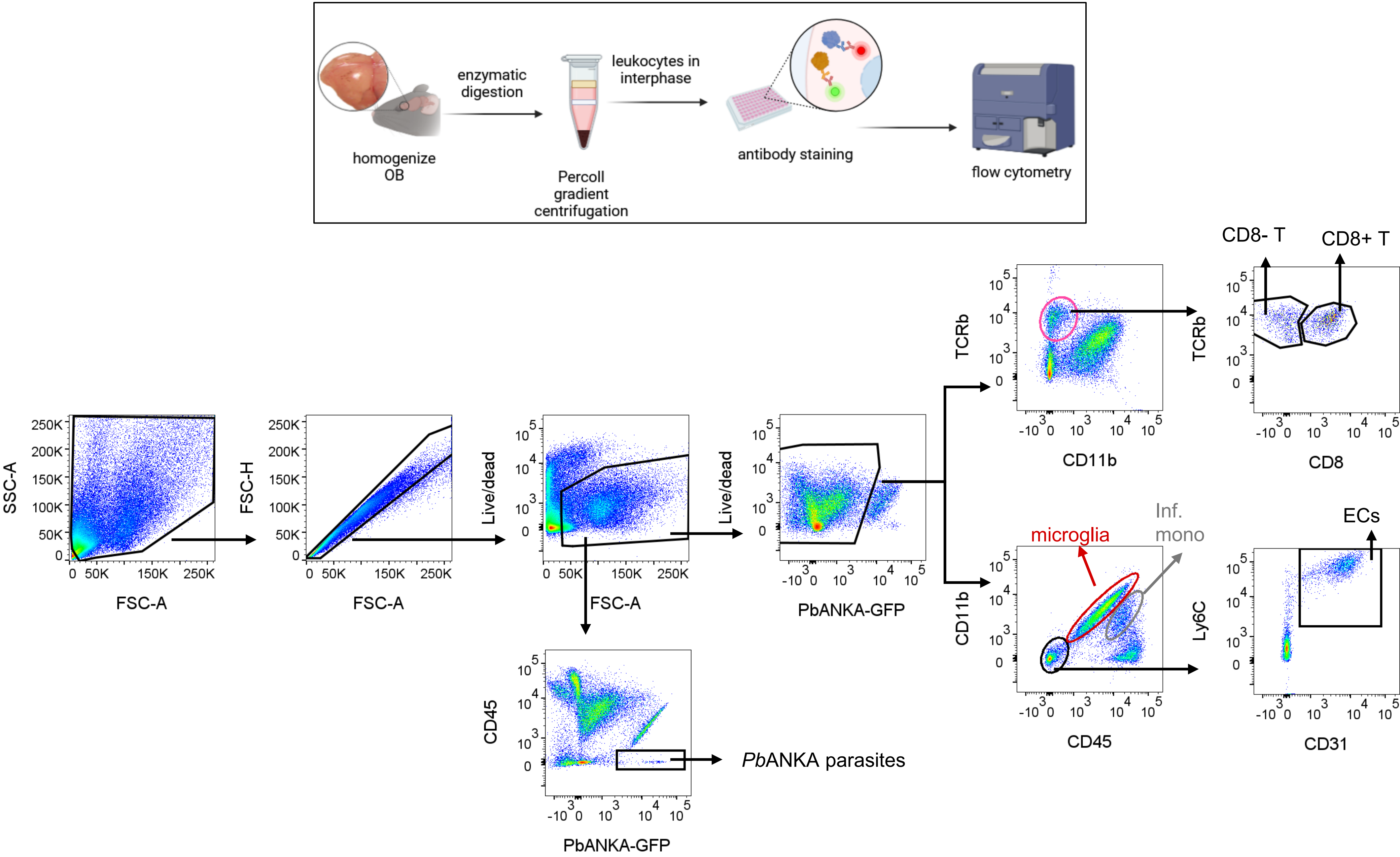

Supplementary Figure 8. Flow cytometry gating strategy for analysis of OB-infiltrating immune cells for data shown in Fig 4.

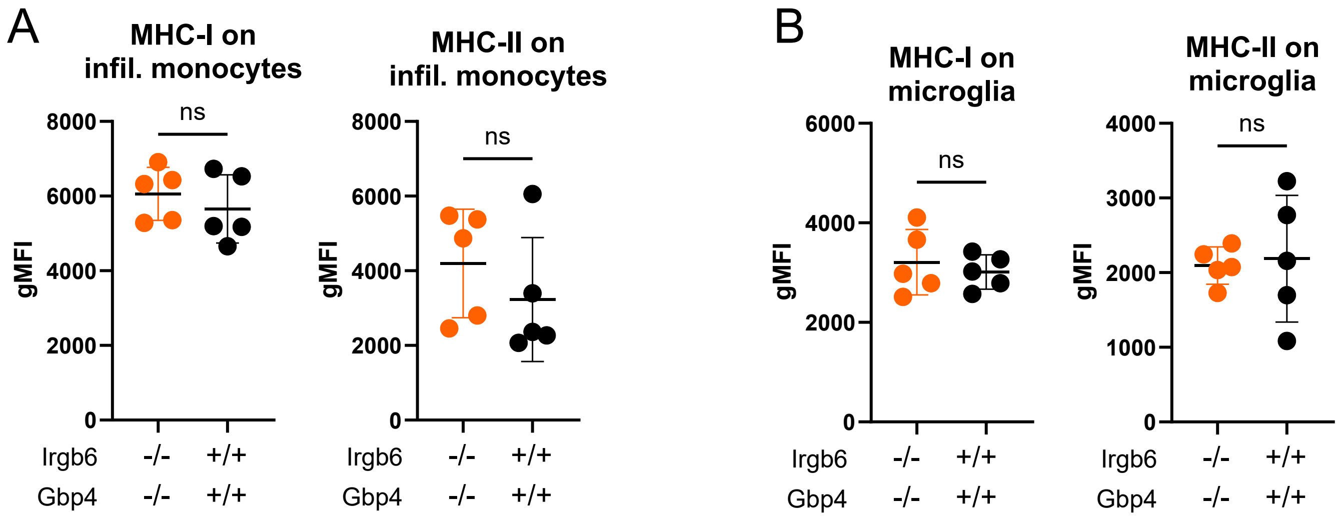

Supplementary Figure 9. MHC-I and -II expressions of infiltrating monocytes **(A)** and microglia **(B)** shown as geometric mean fluorescence intensity (gMFI), in the olfactory bulb of WT and *Irgb6*$^{-/-}$ *Gbp4*$^{-/-}$ mice at day 8 post-infection.

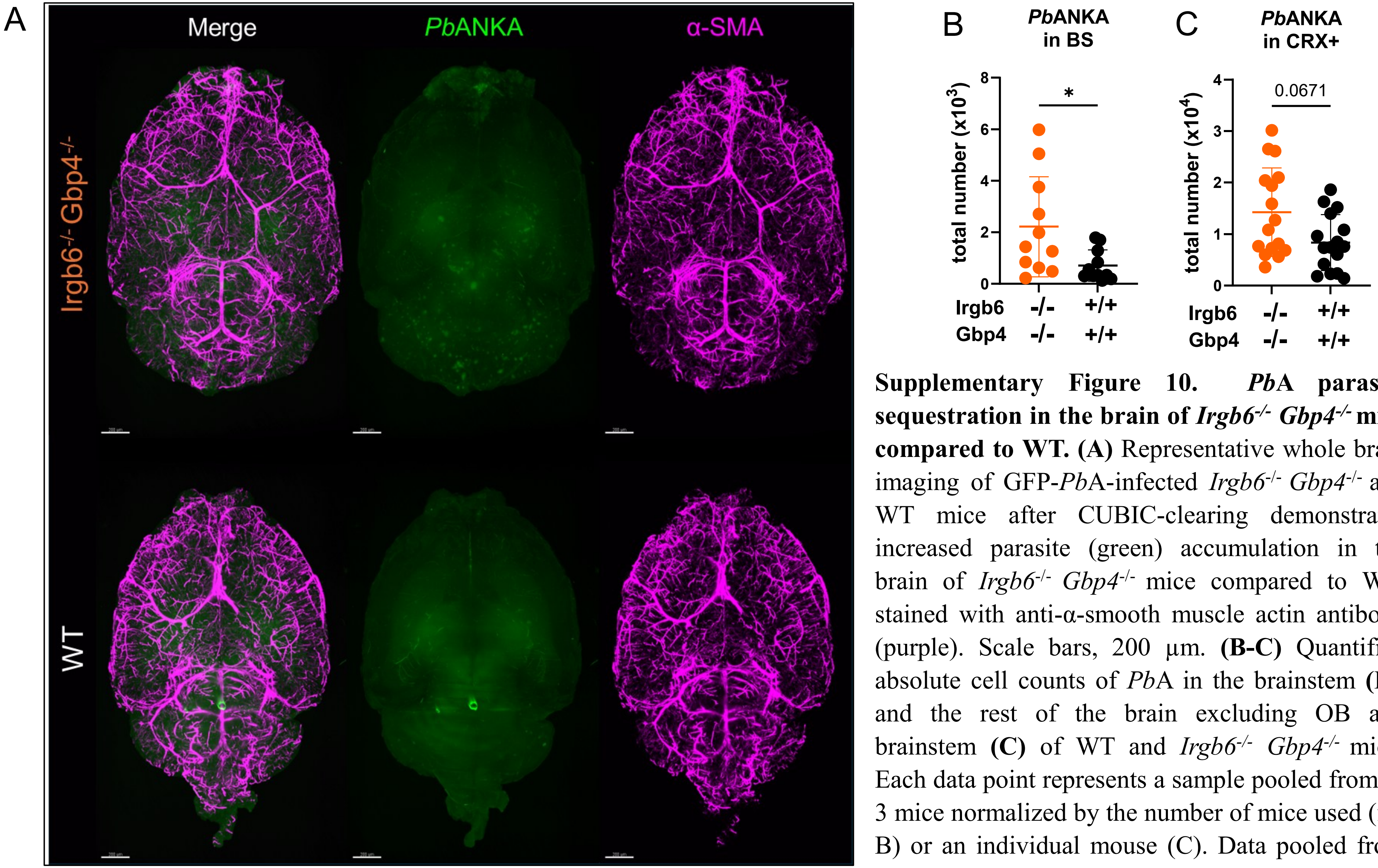

Supplementary Figure 10. *Pb*A parasite sequestration in the brain of *Irgb6*$^{-/-}$ *Gbp4*$^{-/-}$ mice compared to WT. (A) Representative whole brain imaging of GFP-*Pb*A-infected *Irgb6*$^{-/-}$ *Gbp4*$^{-/-}$ and WT mice after CUBIC-clearing demonstrates increased parasite (green) accumulation in the brain of *Irgb6*$^{-/-}$ *Gbp4*$^{-/-}$ mice compared to WT, stained with anti-α-smooth muscle actin antibody (purple). Scale bars, 200 μm. **(B-C)** Quantified absolute cell counts of *Pb*A in the brainstem **(B),** and the rest of the brain excluding OB and brainstem **(C)** of WT and *Irgb6*$^{-/-}$ *Gbp4*$^{-/-}$ mice. Each data point represents a sample pooled from 2-3 mice normalized by the number of mice used (for B) or an individual mouse (C). Data pooled from three independent experiments and shown as mean ± SD. Mann-Whitney test was used for statistical analysis between groups. *, $P<0.05$.

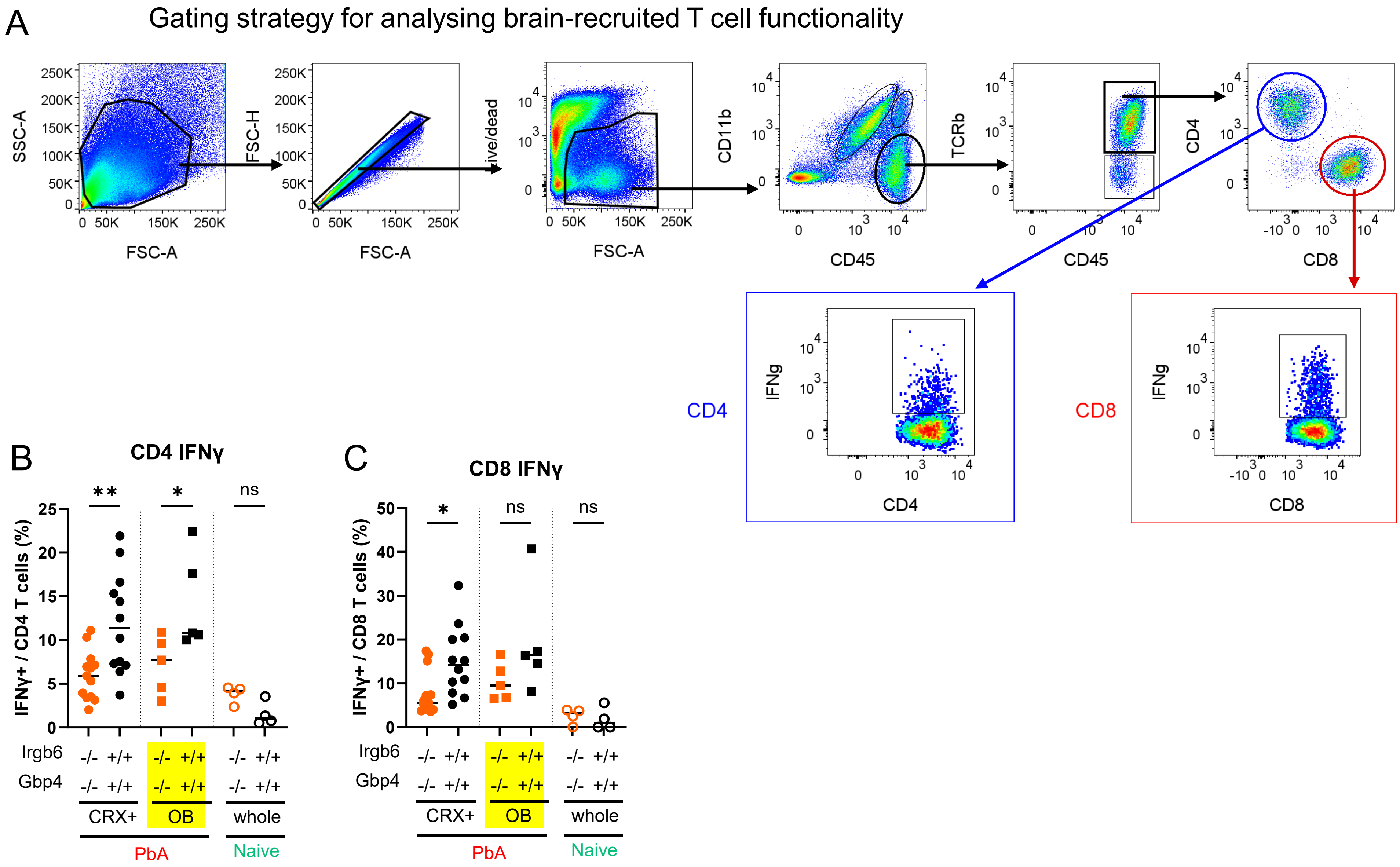

Supplementary Figure 11. Functionality of T cells recruited to brain. (A) Gating strategy for brain T cell functionality as shown in Fig. 5. **(B)** Brain CD4 T cells population and their production of IFN-γ. **(C)** Brain CD8 T cells population and their production of IFN-γ. Highlighted data already shown in main figure, Figure 5.
